# KCNH6 channel promotes insulin exocytosis via interaction with Munc18-1 independent of electrophysiological processes

**DOI:** 10.1007/s00018-024-05134-1

**Published:** 2024-02-13

**Authors:** Hao Wang, Qi Li, Ying-Chao Yuan, Xue-Chun Han, Yong-Ting Cao, Jin-Kui Yang

**Affiliations:** 1 Beijing Key Laboratory of Diabetes Research and Care, Department of Endocrinology and Metabolism, Beijing Diabetes Institute, Beijing Tongren Hospital, Capital Medical University, Beijing, 100730 China; 2 Laboratory for Clinical Medicine, Capital Medical University, Beijing, 100069 China; 3 Department of Endocrinology, Beijing Mentougou District Hospital, Beijing, 102399 China

**Keywords:** KCNH6, Insulin exocytosis, Munc18-1, Kv channel, Insulin secretory granule

## Abstract

Glucose-stimulated insulin secretion (GSIS) in pancreatic islet β-cells primarily relies on electrophysiological processes. Previous research highlighted the regulatory role of KCNH6, a member of the Kv channel family, in governing GSIS through its influence on β-cell electrophysiology. In this study, we unveil a novel facet of KCNH6's function concerning insulin granule exocytosis, independent of its conventional electrical role. Young mice with β-cell-specific KCNH6 knockout (βKO) exhibited impaired glucose tolerance and reduced insulin secretion, a phenomenon not explained by electrophysiological processes alone. Consistently, islets from KCNH6-βKO mice exhibited reduced insulin secretion, conversely, the overexpression of KCNH6 in murine pancreatic islets significantly enhanced insulin release. Moreover, insulin granules lacking KCNH6 demonstrated compromised docking capabilities and a reduced fusion response upon glucose stimulation. Crucially, our investigation unveiled a significant interaction between KCNH6 and the SNARE protein regulator, Munc18-1, a key mediator of insulin granule exocytosis. These findings underscore the critical role of KCNH6 in the regulation of insulin secretion through its interaction with Munc18-1, providing a promising and novel avenue for enhancing our understanding of the Kv channel in diabetes mechanisms.

**Supplementary Information:**

The online version contains supplementary material available at 10.1007/s00018-024-05134-1.

## Introduction

Glucose-stimulated insulin secretion (GSIS) in pancreatic islet β-cells is a tightly regulated process involving various electrophysiological mechanisms, including depolarization via K_ATP_ potassium channels and repolarization mediated by voltage-dependent potassium (Kv) channels. Kv channels are purported to account for the repolarization of pancreatic islet β-cell action potentials, and this has long been believed to be their major contribution to the regulation of insulin secretion [1]. When β-cells are stimulated by high glucose and cause membrane potential depolarization, the Kv channels open to mediate potassium outflow, repolarizes and hyperpolarizes the membrane potential, and regulates insulin secretion by mediating β cell excitability and intracellular calcium concentration [2–4]. Recently, we reported one of the Kv channels, KCNH6 (also called Kv11.2 or hERG2), regulating insulin secretion through its electrical function by studying a large four-generation family with monogenic diabetes [5]. We found that both patients with KCNH6 p.P235L heterozygous mutation and KCNH6 mutant including P235L knock in (KI) and KCNH6 global knockout (GKO) mice had a phenotype from hyperinsulinemia to hypoinsulinemia and diabetes [5]. Interestingly, to better investigate the function of KCNH6 in pancreatic β cells, we generated KCNH6 β cell-specific knockout (βKO) mice recently and found that consistently exhibited a phenotype of impaired glucose tolerance and insulin secretion even before young age (less than 8 weeks) which was different from the phenotype of KCNH6 GKO mice at early adulthood (less than 8 weeks) [5, 6], it seems KCNH6 has some other roles in the regulation of insulin secretion besides its electric function. Therefore, the role of KCNH6 in insulin secretion remains unclear.

In pancreatic β-cells, exocytosis plays a critical role in cellular processes as the last step of insulin secretion. Insulin secretory granule (ISG) exocytosis is triggered by Ca^2+^ influx after opening of voltage-dependent Ca^2+^ channels (VDCCs) [7]. VDCC gating is regulated by changes in membrane potential which is modulated mainly by ATP-sensitive K^+^ (K_ATP_) and voltage-gated K^+^ (Kv) channels [8, 9]. When pancreatic islet β-cells are exposed to high glucose, K_ATP_ channels closure by high ATP concentration results in membrane depolarization, activates VDCC opening, and triggers insulin exocytosis [10]. Conversely, membrane depolarization-induced Kv channels opening leads membrane repolarization, VDCC closure, and termination of insulin exocytosis [4]. In addition to Ca^2+^ influx, the exocytosis of insulin granules is also regulated by the soluble N-ethylmaleimide-sensitive factor attachment protein receptor (SNARE) proteins and their binding partner Munc18 proteins [11, 12]. The SNARE proteins, including syntaxin-1 (STX1), synaptosomal-associated protein 25 (SNAP25) and synaptobrevin 2 (VAMP2), promote granule exocytosis by forming a heterotrimeric complex [13, 14]. It has been reported that Kv2.1 channel directly binds with syntaxin-1a to regulate insulin exocytosis [15]. These reports support the notion that Kv channels do not only regulate membrane repolarization by modulating the ion channels but also participate in the procedure of insulin granule exocytosis through interaction with SNARE proteins.

As the binding partner of SNARE proteins, Munc18 proteins are essential regulators of SNARE protein-mediated vesicle docking or fusion events, acting as high-affinity binding partners for syntaxin proteins [11]. Three plasma membrane-localized Munc18 proteins Munc18-1, Munc18-2, and Munc18-3 are expressed in mammalian cells and have been found to play specific roles in β cell insulin secretion and to modulate glucose homeostasis. Depletion of any of these proteins causes defective GSIS in mouse and human pancreatic islets, whereas overexpression of either Munc18-1 or Munc18-2 potentiates GSIS in human islets [16–18]. Conversely, down-regulation of Munc18 proteins has been observed in pancreatic islets of insulin-resistant rats and patients with T2D [19, 20], suggesting a positive association between islet Munc18 expression and islet function in both rodents and humans. Although the pivotal function of these proteins in insulin secretion is irrefutable, the regulatory mechanism of SM proteins, especially their diminution during T2D, remains ambiguous. Therefore, deciphering the mechanism that regulates Munc18 proteins expression or activity is of paramount importance to understand the pathophysiology of T2D, paving the way for the generation of antidiabetic therapies. However, the relationship between Munc18 proteins and Kv channels has not been clarified.

In this study, we explored a novel mechanism by which KCNH6 regulates β cell insulin exocytosis through interaction with the exocytotic protein Munc18-1. We found that young KCNH6 β cell-specific knockout (βKO) mice showed impaired glucose tolerance and lower levels of serum insulin. KCNH6 deficient β cells strongly reduced GSIS. Results from electron microscopy and total internal reflection fluorescence microscopy (TIRFM) suggested that KCNH6 is required for insulin vesicle docking and two modes of ISG fusion. We further demonstrated that KCNH6 regulates β cell insulin secretion through interaction with exocytotic proteins Munc18-1. Disruption of native KCNH6-Munc18-1 interaction by a KCNH6 3A mutant resulted in defective glucose-stimulated insulin secretion, whereas restoration of KCNH6 expression rescues impaired insulin secretion. Our findings suggest that KCNH6/Munc18-1 complex plays a key role in insulin exocytosis.

## Materials and methods

### Animal model and phenotypic characterization

C57BL/6J mice were purchased from Vital River Laboratories (Beijing, China). The KCNH6 β-cell-specific knockout (KCNH6-βKO) mice were generated as described previously [6]. The mice were housed at constant temperature and humidity, with a 12 h light and dark cycle, and fed a regular unrestricted diet. Only male mice and their tissues and cells were phenotypically characterized in this study. Phenotypic analyses of mice were performed as described previously [21]. Animal experiments followed the national ethical guidelines implemented by our Institutional Animal Care and Use Committee and were approved by the Ethical Review Committee of the Institute of Zoology, Capital Medical University, China.

### Cell culture and glucose-stimulated insulin secretion assay

MIN6 cells [22] were cultured in high glucose Dulbecco's modified Eagle's medium (DMEM) containing 15% fetal bovine serum, 50 µM 2-mercaptoethanol and 1% penicillin–streptomycin. Human embryonic kidney (HEK) 293A cells (Cell Resource Center, Chinese Academy of Medical Sciences, Beijing, China) were cultured in high glucose DMEM containing 10% FBS and 1% penicillin–streptomycin. Pancreatic islets were isolated from mice through injection of 500 units/mL collagenase solution (type XI; Sigma) into the pancreatic duct, followed by mild shaking digestion at 37 °C for 20 min, and isolated islets were manually selected under a dissecting microscope, as described elsewhere [23]. Isolated islets were cultured overnight in RPMI 1640 medium containing 10% FBS and 1% penicillin–streptomycin. After overnight recovery, islets were treated with 0.05% trypsin in PBS at 37 °C for 4 min, fully dispersed to primary β-cells. All cells were cultured in a humidified incubator with 95% air and 5% CO_2_ at 37 °C. Glucose-stimulated insulin secretion assay in MIN6 cells and perifused islets was performed as described previously [21]. For experiments with pharmacologic treatments, 5 μM Berberine (Sigma-Aldrich, St. Louis, MO, USA) was used as described previously [6].

### Subfractionation

Subcellular fractions of MIN6 cells were performed on ice. Plasma membrane (PM) proteins were isolated using the MinuteTM Plasma Membrane Protein Isolation Kit (SM-005, Invent Biotechnologies, Plymouth, MN, USA). To get granules fraction, cells were scraped in 1 ml homogenization buffer (20 mM HEPES, pH 7.4; 0.5 mM EDTA; 0.5 mM EGTA; 250 mM sucrose and 1 mM dithiothreitol) containing protease inhibitors (Roche). The cells were then disrupted by 10 strokes through a 26 G needle. The cell homogenates were centrifuged 900 ×*g* for 10 min to remove the nuclei and unbroken cells. Post-nuclear supernatant was centrifugated at 5500 ×*g* for 15 min and then at 25 000 ×*g* for 20 min to pellet the granule fraction. The supernatant was further centrifugated at 100,000 ×*g* for 1 h to obtain the cytosolic fraction (supernatant).

### Antibodies and immunoprocedures

In these studies, our novel (noncommercial) polyclonal antibodies against amino acids 20–38 (RKFEGQSRKFLIANAQMEN) of the mouse KCNH6 protein (mKCNH6-Ab) were conjugated with carrier protein KLH for immunization of rabbits. This antibody was customized at PTM Biolabs (Hangzhou, China) and validated by enzyme-linked immunosorbent assay and western blotting. The sources of antibodies and their concentrations used are listed in Supplementary Table 1. All Immunoprocedure experiments were performed as previously described [24]. Islets or cells were lysed in lysis buffer containing 20 mM Tris–HCl pH 7.5, 150 mM NaCl, 1 mM MgCl_2_, 10 mM EGTA, 1% Triton X-100, 1 mM PMSF and a complete protease inhibitor cocktail (Roche). Protein samples from lysates were separated by SDS-PAGE and then transferred to a polyvinylidene difluoride membrane (Millipore). The membrane was blocked with TBST (TBS plus 0.1% Tween-20) containing 0.5% nonfat dried milk powder and then incubated overnight at 4 °C with the primary antibody followed with incubation of horseradish peroxidase-conjugated secondary antibody (1:2000, Beyotime, China) for 1 h at room temperature, and was washed five times. The immunoreactive bands were detected using enhanced chemiluminescence (Amersham Biosciences, USA) and an LAS-500 chemiluminescence detection system (GE Healthcare Bioscience, USA). Optical density values of immunoreactive bands were obtained using Image J software. For immunoprecipitation, cell lysates were performed at 4 °C by incubation with primary antibody overnight, followed by the addition of Protein G-Sepharose 4F (GE Healthcare Bioscience) for 1 h or by direct incubation with anti-hemagglutinin (HA) affinity matrix beads (Roche Diagnostics) or anti-FLAG affinity gel (Sigma-Aldrich) for 1 h. After being washed five times with wash buffer containing 20 mM Tris–HCl pH 7.5, 150 mM NaCl, 10% Glycerol, 0.1% TritonX-100, the immunoprecipitates were subjected to SDS-PAGE. For immunofluorescence, primary β-cells cultured on poly-L-lysine-coated 35-mm glass base dishes (150680, Thermo, USA) were fixed by 4% paraformaldehyde for 30 min at room temperature and were permeabilized with 0.1% Triton X-100 in PBS for 30 min, then blocked with PBS containing 1% bovine serum albumin (BSA) for 30 min. The coverslips were incubated with primary antibodies overnight at 4 °C, followed by incubation of Alexa Fluor 488- or 568-conjugated secondary antibodies (Invitrogen, USA) for 1 h at room temperature. After being washed five times with PBS, samples were mounted using a mounting solution containing DAPI (for nuclear staining) reagent (Beyotime, Shanghai, China). Confocal imaging was performed using an FV-3000 (Olympus) confocal laser scanning microscope equipped with a 100 × oil immersion objective lens (1.45 NA) and FV31S-SW. The images were adjusted using FV31S-SW and Image J software. Each image is representative of at least three independent experiments.

### DNA and RNA manipulation

Mouse KCNH6 and Munc-18-1 cDNAs were derived from MIN6 cells. Point and deletion mutants were generated using a standard PCR-based mutagenesis strategy and were verified by DNA sequencing. The sequences of the primers used are listed in Supplementary Table 2. These cDNAs were subcloned into pcDNA3-HA, pcDNA3-FLAG vector (Invitrogen) or mCherry-C1 vector (Clontech) as described previously [24]. Insulin-EGFP was generated as described previously [25]. To generate recombinant adenoviruses, KCNH6 WT and KCNH6 R246A/T248A/L250A (3A) mutant were inserted into pENTR-3C (Invitrogen) and were transferred into pAd/CMV by LR Clonase recombination (Invitrogen), which co-produces red fluorescent protein (Cherry) to allow identification of transfected cells. To express an exogenous protein, HEK293A cells were transfected with the plasmids using Lipofectamine 3000 reagent (Invitrogen), whereas MIN6 cells were infected with adenoviruses.

### RT-qPCR assay

Total RNA was extracted from islets using Trizol reagent (Thermo Fisher Scientific, USA) following the instructions of the manufacturer. Next, the first-strand cDNA was synthesized from an RNA template using SuperScriptIII Reverse Transcriptase (Thermo Fisher Scientific). Subsequently, quantitative PCR reactions were performed using TransStart Tip Green qPCR SuperMix (TransGen Biotech, Beijing, China), cDNA template, and specific PCR primers. Rplp0/36B4 acted as the housekeeping gene to normalize the expression of other genes. The quantitative PCR primer sequences were presented in Supplementary Table 3.

### Intracellular calcium level measurement

Primary murine pancreatic β-cells were plated on glass coverslips and left in a humidified incubator for 24 h. Before the imaging experiment, cells were loaded with 2 μM Fluo-4 AM (Dojindo, Japan) in a Krebs–Ringer bicarbonate buffer (KRBB; 120 mM NaCl, 5 mM KCl, 24 mM NaHCO_3_, 1 mM MgCl_2_, 2 mM CaCl_2_, 15 mM HEPES pH 7.4, 0.1% BSA) with 2.8 mM glucose (low glucose) at 37 °C for 30 min, then washed twice using low-glucose KRBB without Fluo4-AM. Afterwards, cells were pre-incubated with low-glucose KRBB for another 30 min and then were stimulated with 16.7 mM high-glucose KRBB. Experiments were performed using a DeltaVision Ultra High Resolution Microscope (GE, USA) with a 60 × objective. Fluo 4-AM was excited at 488 nm and emission was collected at 525 nm. Sequential images of cells were recorded starting from 60 s before stimulation at 5 s internals and analyzed using an Image J software. The ratio of fluorescence change *F*/*F*0 was used to reflect changes in intracellular Ca^2+^ levels, where *F* is the observed fluorescence density, and *F*0 is the average value of baseline fluorescence density of the first 30 s before stimulation.

### Total internal reflection fluorescence microscopy

Isolated islets were dissociated into single cells by incubation with trypsin–EDTA solution as described previously [24]. The dispersed cells were cultured on poly-L-lysine-coated 35-mm glass base dishes (150680, Thermo, USA) for 2 days. A monolayer of the cells was infected with adenovirus encoding enhanced green fluorescent protein (EGFP)-tagged human preproinsulin and further cultured for 2 days. TIRF microscopy was performed using an inverted microscope Eclipse with a 1.49-numerical aperture objective lens ApoTIRF100 × (Nikon). Images were acquired at 101 ms intervals with an electron multiplying charge-coupled device camera iXon DU-897 (Andor Technology). The cells were preincubated for 30 min in low-glucose KRBB at 37 °C, and after image recording for 30 s, they were exposed to glucose stimulation for 20 min. The stimulation was achieved by the addition of the same volume of twofold concentrated solution, resulting in a final concentration of 25 mM glucose. Fusion events with a flash were manually selected and assigned to one of three types: *residents*, which are visible at least for 10 s before fusion; *visitors*, which have become visible during stimulation and remained visible for more than one consecutive frame before fusion; and *passengers*, which are visible for less than one frame (101 ms) before fusion [24]. The average fluorescence intensity of individual vesicles was calculated as described previously [24].

### Transmission electron microscopy

Islets were fixed with 2.5% (vol/vol) glutaraldehyde with Phosphate Buffer (PB) (0.1 M, pH 7.4), washed four times in PB at 4 °C. Then islets were postfixed with 1% (wt/vol) OsO_4_ and 1.5% (wt/vol) potassium ferricyanide aqueous solution at 4 °C for 2 h, dehydrated through a graded ethanol series (30, 50, 70, 80, 90, 100%, 100%, 5 min) into pure acetone (2 × 5 min). Samples were infiltrated in a graded mixture (3:1, 1:1, 1:3) of acetone and SPI-PON812 resin (21 ml SPI-PON812, 13 ml DDSA and 11 ml NMA), then changed to pure resin. Finally, samples were embedded in pure resin with 1.5% BDMA and polymerized for 12 h at 45 °C, 48 h at 60 °C. The ultrathin Sections (70 nm thick) were sectioned with a microtome (Leica EM UC6), double-stained by uranyl acetate and lead citrate, and examined by a transmission electron microscope (FEI Tecnai Spirit120kV). The number of mature and immature secretory granules, cell size and the distance from the plasma membrane to secretory granules were manually counted and quantified using FIJI (ImageJ) software [26]. A distance < 200 nm from the center of the granule to the plasma membrane was considered to be a docked granule.

### Patch-clamp experiments

Whole-cell patch-clamp experiments were performed as previously described [5]. Data were collected using PatchMaster v2 × 80 software. For recording KCNH6 channel currents, transfected HEK293 cells were elicited by 3 s depolarizing pulses ranging from − 60 to + 60 mV and the tail currents by 2 s repolarizing pulses to − 40 mV. The bath solution solution contains (in mM) NaCl 137, KCl 4, CaCl_2_ 2, MgCl_2_ 1, Glucose 10, HEPES 10 and was adjusted to pH 7.3 with NaOH. The pipettes solution contains (in mM) KCl 130, MgATP 5, MgCl_2_ 1, EGTA 5, HEPES 10 and was adjusted to pH 7.3 with KOH.

### Molecular docking

We used the HDOCK server (http://hdock.phys.hust.edu.cn/) to carry out protein–protein molecular docking. HDOCK is a powerful pipeline for integrated protein–protein docking, which is based on a hybrid docking algorithm of template-based modeling and ab initio-free docking to optimize the adjustment of ligand [27, 28]. HDOCK performed rigid-body docking using an FFTW-based hierarchical approach by mapping the receptor and ligand molecules onto grids and incorporated experimental information on protein–protein binding sites and small-angle X-ray scattering during the docking and post-docking processes and simplicity of use. In this study, the 3D structures of KCNH6 (B1AR82_MOUSE) and Munc18-1 (STXB1_MOUSE) were downloaded from the AlphaFold Protein Structure Database (https://alphafold.com/) [29]. We used KCNH6 as a receptor and Munc18-1 as a ligand, and submitted to the HODCK server with default parameters. The output model with the highest score was selected and visualized using the program PyMol (https://pymol.org/2/).

### Statistical analysis

All statistical analyses were conducted with the software GraphPad Prism version 8.0 Data are presented as means ± SEM. Statistical significance was determined using the Student *t* test and one-way ANOVA with a Tukey’s test. A *p* value of < 0.05 was considered significant.

## Results

### Young KCNH6-βKO mice showed impaired glucose tolerance and decreased insulin secretion

**Fig. 1:**
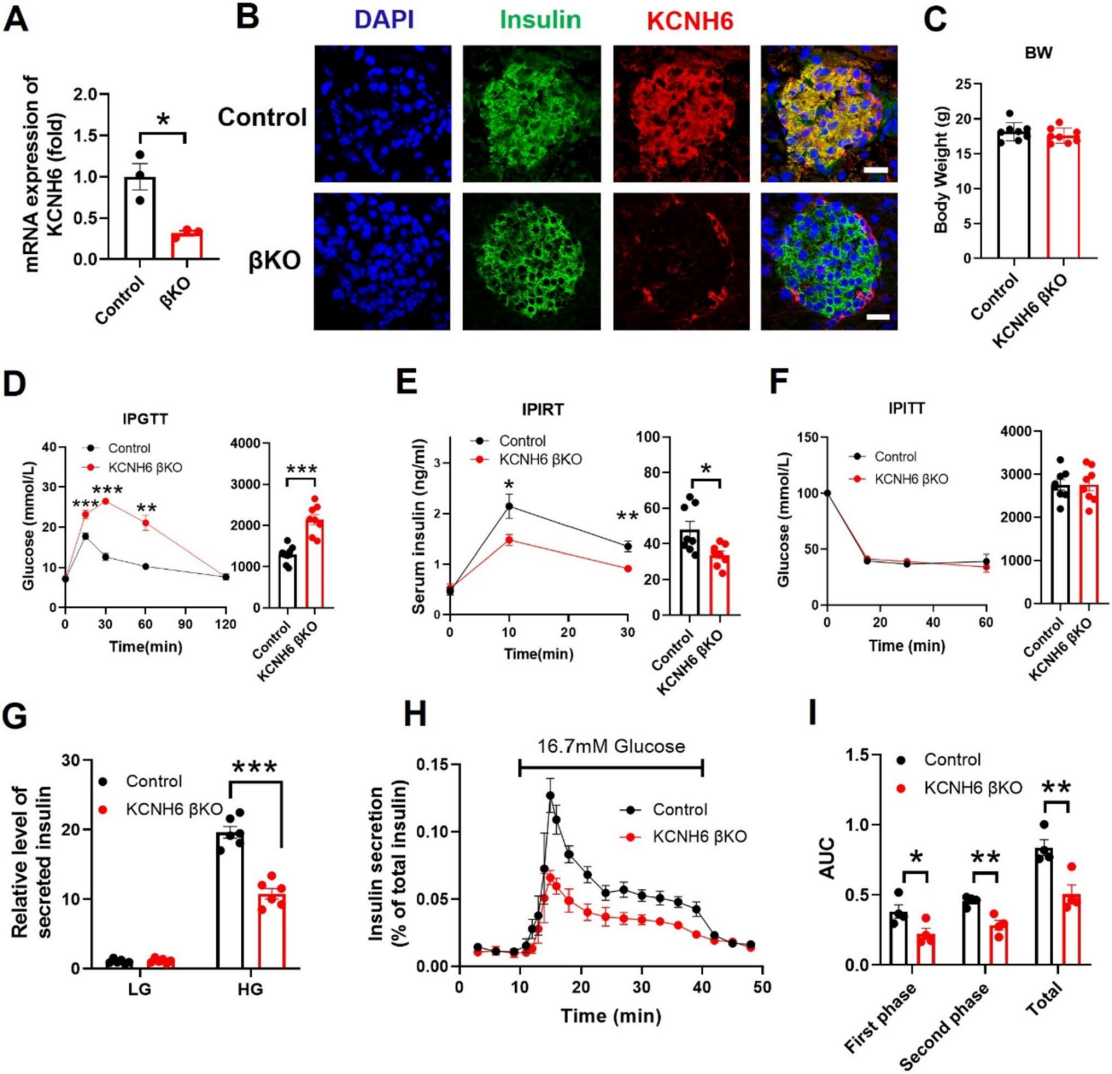
KCNH6-βKO mice showed impaired glucose tolerance and decreased insulin secretion. **A** KCNH6 mRNA expression in WT and KCNH6-βKO mouse islets (*n* = 3). **B** Immunofluorescence staining for localization of insulin and KCNH6 expressed in WT and KCNH6-βKO mouse islets. Nuclei were counterstained with DAPI, which appears blue. KCNH6 is in red, and insulin is in green. All Bars in white are 20 μm in length. **C** Body weight (BW) comparison between WT and KCNH6-βKO mice. **D** Blood glucose levels and area under the curve (AUC) during an intraperitoneal glucose tolerance test (IPGTT) (2 g glucose/kg bw) (*n* = 8 for each). **E** Serum insulin concentration and AUC to IPGTT. **F** Consecutive blood glucose levels and AUC during intraperitoneal insulin tolerance test (IPITT) (0.75 units insulin/kg bw) (*n* = 8 for control; *n* = 8 for KCNH6-βKO). **G** Insulin secretion assay in WT and KCNH6-βKO islets (*n* = 6). **H**, **I** Approximately 30 islets from WT and KCNH6-βKO mice were perfused with a low (2.8 mM) glucose concentration for 10 min followed by a high (16.7 mM) glucose concentration for 30 min. **H** The amount of secreted insulin was normalized to the total insulin content. **I** First phase (0–5 min), second phase (6–30 min) and total insulin secretion (1–30 min) were calculated as the AUC. (*n* = 4). *p < 0.05, **p < 0.01, ***p < 0.001 by Student *t* test

Our previous study reported that KCNH6 global knockout (GKO) mice exhibited early-stage hyperinsulinemia and hypoinsulinemia in later adulthood [5]. Since KCNH6 was widely expressed in metabolic-related organs [5, 6], to further elucidate the function of KCNH6 in pancreatic β cells, we used the Cre-LoxP recombinase system to generate pancreatic islet β-cell-specific KCNH6 knockout (βKO) mice [6]. However, we found that KCNH6-βKO mice exhibited severe phenotype of impaired glucose tolerance and insulin secretion even at 8 weeks age which was different from those of KCNH6 GKO mice [5, 6]. To confirm this phenotype, we intended to investigate the phenotype of KCNH6-βKO mice at an early age again. We first verified the lack of KCNH6 in β-cells in KCNH6-βKO mice. The mRNA level of KCNH6 was significantly reduced in islets isolated from KCNH6-βKO mice compared to age-matched control mice (Fig. 1A). Loss of KCNH6 did not affect the mRNA expression level of other KCNH channels and some important Kv channels (Figure S1). Meanwhile, immunofluorescence staining results proved the lack of KCNH6 in insulin-produced β-cells of KCNH6-βKO mice (Fig. 1B). Then, we analyzed phenotypes of 6-weeks-old age-matched KCNH6-βKO and control mice. The body weight of KCNH6-βKO mice and WT mice showed no difference (Fig. 1C). Interestingly, KCNH6-βKO mice exhibited impaired glucose tolerance (Fig. 1D) and lower serum insulin concentrations (Fig. 1E) after glucose loading which was different from those of KCNH6 GKO mice [5]. Furthermore, KCNH6-βKO mice had normal insulin sensitivity (Fig. 1F), which indicated that the impaired glucose tolerance in KCNH6-βKO mice was associated with a decrease in insulin secretion capacity. To gain deeper insights into the involvement of KCNH6 in insulin secretion, we conducted a glucose-stimulated insulin secretion (GSIS) test using isolated islets from 6-week-old KCNH6-βKO mice and control mice. A significant reduction in insulin secretion was observed in the KCNH6-βKO islets as compared to the control islets (Fig. 1G). This phenomenon of impaired insulin secretion from KCNH6-βKO islets was consistently observed across KCNH6-βKO mice of different ages (Figure S2). Notably, the amplitude and time course of the glucose-induced rise in the cytoplasmic Ca^2+^ concentration were similar between pancreatic β cells isolated from control and KCNH6-βKO mice (Figure S3). We next assessed the insulin-secretory ability of isolated islets from 6-week-old KCNH6-βKO mice by perifusion analyses. KCNH6 deficient islets showed a biphasic decreased glucose-induced insulin secretion as compared to that of control islets (Fig. 1H). The area under the curve showed that both the first (1–5 min) and second phase (6–30 min) of insulin secretion were significantly reduced in KCNH6 deficient islets (Fig. 1I). These findings suggested that KCNH6-βKO mice showed glucose intolerance and impaired insulin secretion even at an early age, which is opposed to the phenotype identified in GKO mice. Therefore, we hypothesized that KCNH6 might have some other effect on insulin secretion independent of electrophysiological processes.

### KCNH6 was partially expressed on insulin granules and promoted biphasic insulin secretion

**Fig. 2:**
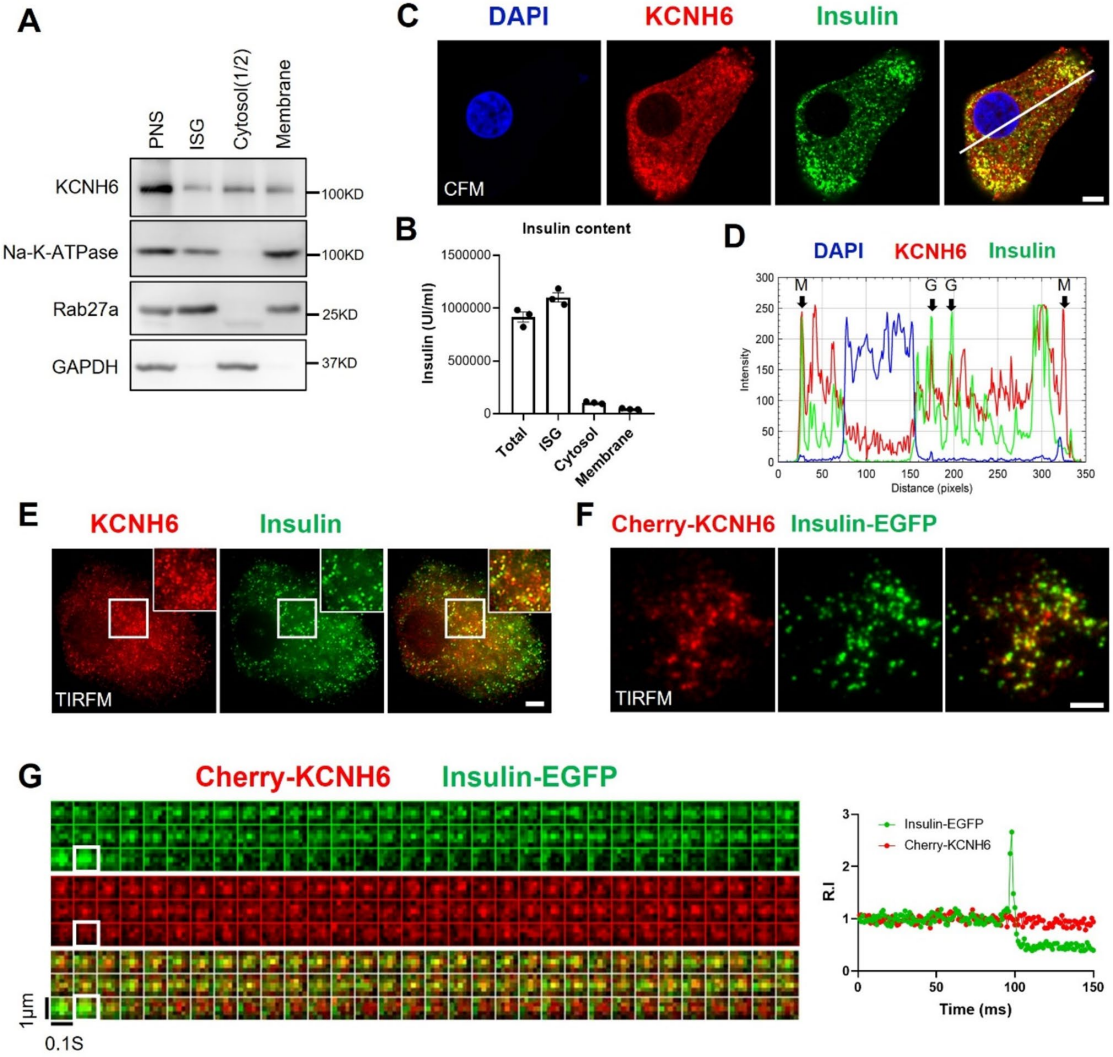
KCNH6 was located on insulin granules and associated with insulin granule fusion. **A** KCNH6 expression was examined in different cellular fractions of mouse β cells including insulin granules, cytoplasm, and cell membrane fractions. Na–K-ATPase protein was used as a marker for the cell membrane, Rab27a protein and insulin content on the right side **B** were used as markers for insulin granules, GAPDH protein was used as a marker for the cytoplasm. **C** Colocalization of KCNH6 with insulin granules was examined using confocal fluorescence microscopy (CFM). **D** Fluorescent intensity profiles along the indicated line of KCNH6 and insulin are shown at right. Arrows indicate the membrane (M) and granule (G) regions. Bar: 10 μm. **E** Colocalization of KCNH6 with insulin granules near the plasma membrane was examined using total internal reflection fluorescence microscopy (TIRFM) in fixed pancreatic beta cells. Bar: 5 μm. **F** An example showing colocalization of Cherry-KCNH6 and SGs (labelled by Insulin-EGFP) in living pancreatic β cells under TIRFM. Bar: 5 μm. **G** Kymographs and corresponding time-lapse relative fluorescence-intensity curves (green, Insulin-EGFP; red, Cherry-KCNH6) of fused insulin granules, white square indicated fusion of ISG. Data shown are representative of three independent experiments for each; *RI* relative intensity

Insufficient insulin release can result from abnormalities in β cell development, ISG biogenesis, insulin production, or exocytosis. To distinguish between these possibilities, we first investigated the localization of KCNH6 through subcellular fractionation (Fig. 2A and B). In addition to crude membrane fraction (including plasma membrane and organelle membrane) and cytosol fraction as described previously [5, 30], we newly found that KCNH6 was expressed in the secretory granule fraction. Immunofluorescence staining further confirmed this observation, showing that KCNH6 was widely expressed in β cell including membrane, cytosol and ISGs (Fig. 2C and D). Results from Total Internal Reflection Fluorescence Microscopy (TIRFM) demonstrated significant colocalization of KCNH6 and ISGs in close proximity to the plasma membrane of the β cell (Fig. 2E). This observation strongly suggests that KCNH6 was partially expressed on insulin granules and might have a direct impact on the process of insulin secretion. This was also the case with living β cells, in which Cherry-KCNH6 fluorescence colocalized with Insulin-EGFP fluorescence (Fig. 2F). We then monitored ISG-associated KCNH6 fluorescence, we found that both green and red fluorescent spots displayed coincident mobility kinetics by recording the movement of Cherry-KCNH6 and Insulin-EGFP under TIRFM (Fig. 2G). These findings demonstrated that KCNH6 was abundant in ISGs in pancreatic beta cells.

### KCNH6 deficient diminished biphasic GSIS by inhibiting resident and passenger type of insulin granule exocytosis

**Fig. 3:**
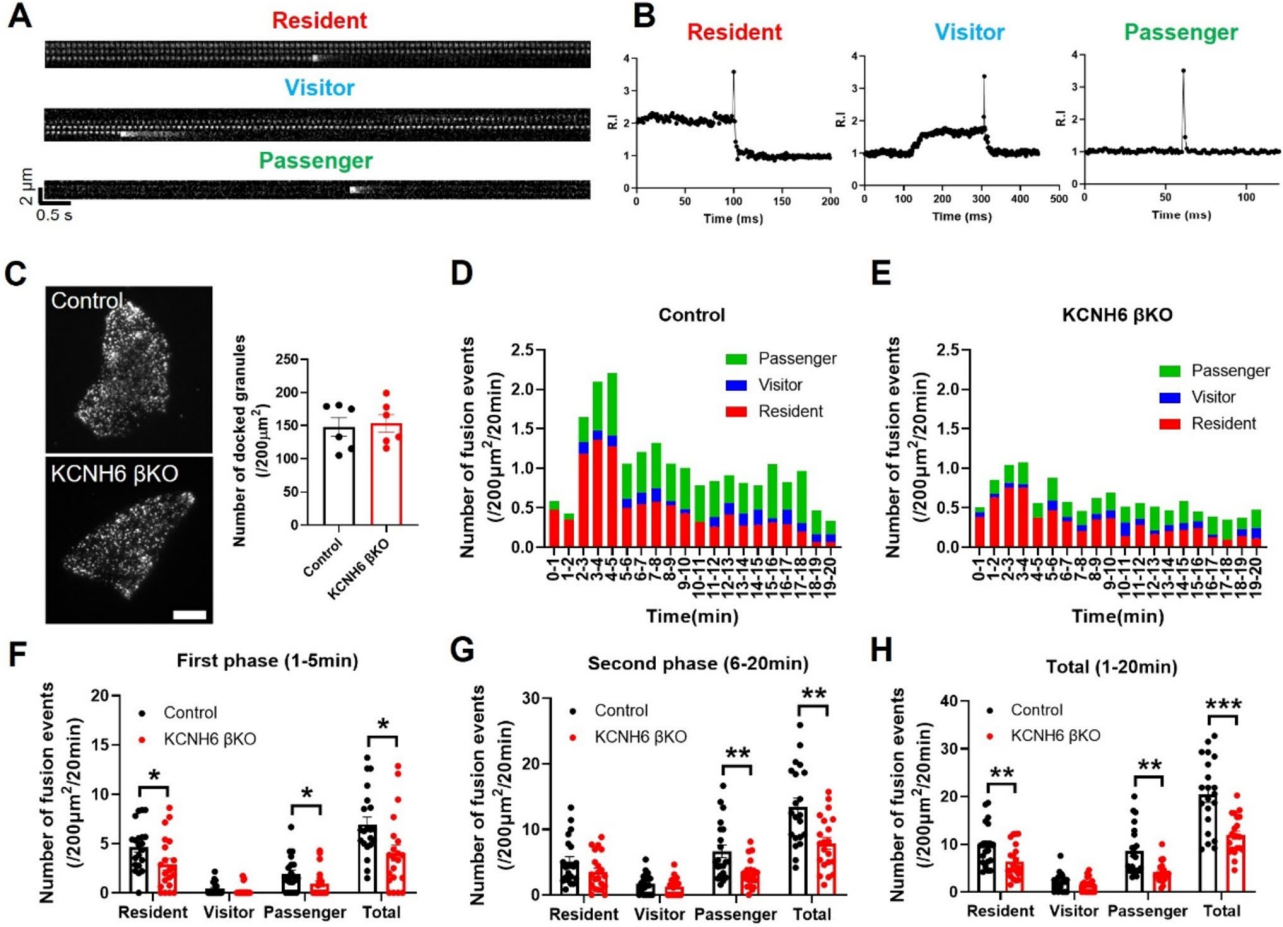
KCNH6 deficient β cells showed reduced biphasic insulin exocytosis after glucose stimulation. **A** The primary pancreatic islet β cells from mice expressing Insulin-EGFP were stimulated with 16.7 mmol/L glucose in KRBB solution. The images shown in the figure represent typical examples of residents, visitors and passengers. The bright white spots indicate the fusion of insulin granules with the plasma membrane. **B** The relative fluorescence intensity for each type of (**A**) was calculated in a 1 μm × 1 μm square around individual vesicles at each time point before and after fusion. **C** Images acquired through TIRFM were captured following a 30-min incubation of primary mouse pancreatic islet β cells expressing Insulin-EGFP in 2.8 mmol/l glucose KRBB and statistical analysis of docked granules (*n* = 6). Bar: 5 μm. **D**–**E** Histogram of the three patterns of fusion events from β cells expressing Insulin-EGFP stimulated with 16.7 mmol/l glucose KRBB: passenger (green bar), visitor (blue bar), and resident (red bar) in control (**D**) and KCNH6 null (**E**) β-cells (*n* = 21). **F**–**H** Summary of the three patterns of fusion events in the first phase (1–5 min) (**F**), second phase (6–20 min) (**G**) and total fusion events (1–20 min) (**H**). *p < 0.05, **p < 0.01, ***p < 0.001 by Student *t* test

Time-lapse imaging recordings under TIRFM in living cells permit direct observation of the fusion events of secretory granules, as well as analysis of the prefusion behavior of fused granules. Since over 90% of the monolayer cells derived from wild-type islets are β-cells [24], we employed TIRF microscopy to monitor the exocytosis of ISGs labeled with Insulin-EGFP just beneath the plasma membrane in living β cells. On stimulation, single IG fusion events were observed as flashes of fluorescence that rapidly dissipated in a cloud-like diffusion pattern. These fused granules were categorized into residents, visitors, and passengers as described previously (Fig. 3A and B) [24, 25]. Under basal state (2.8 mmol/l glucose), the levels of punctuate fluorescence indicating docked ISGs were not different between control and KCNH6-βKO islet cells (Fig. 3C). However, upon high-glucose stimulation (16.7 mmol/l), both residents and passengers contributed to first-phase GSIS and second-phase GSIS respectively. The total number of fusion events during both the first and second phases of glucose stimulation was significantly reduced in KCNH6-βKO β cells as compared to control cells (Fig. 3D–H), which is consistent with the result of the perifusion analysis. In the first-phase GSIS, there was an obvious reduction of residents and passengers from KCNH6 deficient β cells (Fig. 3D–F). In second-phase GSIS, there was a decline only in passengers from KCNH6 deficient β cells (Fig. 3D, E and G). This result suggests that KCNH6 depletion inhibits biphasic GSIS by reducing both resident and passenger types of insulin granule exocytosis.

### KCNH6 affected the replenishment of ISGs docked to the PM in β cells after glucose stimulation

**Fig. 4:**
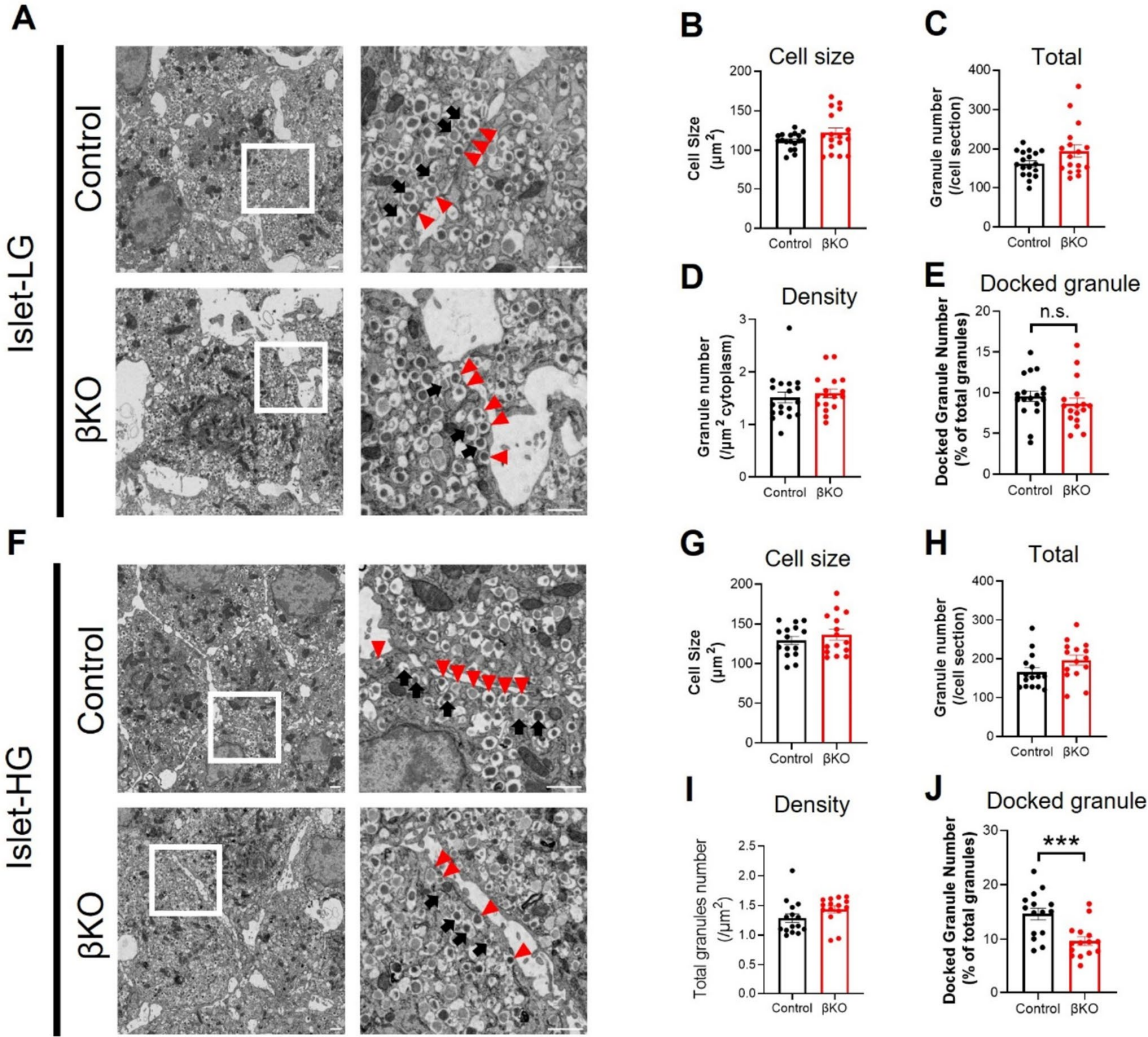
KCNH6 affected replenishment of insulin granules docked to the plasma membrane in islet after glucose load. **A** Insulin granule morphology in Control and KCNH6-βKO β cells under 2.8 mmol/l glucose KRBB (LG) condition (*n* = 18 for control from 3 mice; *n* = 17 for KCNH6-βKO from 3 mice). Squares in the left panels are shown at a higher magnification in the right panels. White arrowheads indicate the granules of the reserve pool, whereas red arrows indicate the granules of the docked pool. Bar, 1 μm. **B**–**D** Cell size (**B**), total number of granules (**C**), and average granule density (**D**) in control and KCNH6-βKO β cells under LG condition. **E** The percentage of docked granules to total granules in control and KCNH6-βKO β cells under LG condition. A distance < 200 nm from the center of the granule to the plasma membrane was considered to be a docked granule. **F** Insulin granule morphology in control and KCNH6-βKO β cells after 16.7 mmol/l glucose KRBB (HG) stimulation (n = 17 for control from 3 mice; *n* = 17 for KCNH6-βKO from 3 mice). **G**–**I** Cell size (**G**), total number of granules (**H**), and average granule density (**I**) in control and KCNH6-βKO β cells after HG stimulation. **J** The percentage of docked granules to total granules in WT and KCNH6-βKO β cells after HG stimulation. ***p < 0.01 by Student t test; n.s. means not significant

Although TIRFM is excellent at examining the behavior of ISGs close to the plasma membrane (PM) within a range of up to 100 nm, we wanted to expand our investigation to assess the behavior of ISGs further inside the cytoplasm, whether ISGs are adequate to replenish reduced docked ISGs near the PM that have been depleted by glucose-stimulated exocytosis. Specifically, we sought to determine whether ISGs are effectively recruited to the PM to replenish the pool of ISGs near the PM that may have been depleted during glucose-stimulated exocytosis. To achieve this, we conducted electron microscopy (EM) morphometric analysis of both control and KCNH6-βKO β cells under basal conditions and after stimulation with 16.7 mmol/l glucose. Under basal conditions, there were no significant differences in cell size, ISG numbers, and docked granule numbers between KCNH6-βKO β cells and those of control β cells (Fig. 4A–E), which aligned with the observations from the TIRFM analyses (Fig. 3C). These findings indicate that KCNH6 does not play a direct role in ISG biogenesis. We then examined the ability of ISGs to mobilize to the PM after glucose-stimulated depletion of the docked ISGs in the releasable pool (RP). With high glucose stimulation, it is expected that a reduced number of docked granules in RP would be replenished to facilitate insulin secretion [31]. Surprisingly, we found that the number of docked granules in KCNH6-βKO β cells did not exhibit the expected increase after stimulation, resulting in a significant reduction compared to control β cells (Fig. 4F and J). This suggests that KCNH6 deficiency impairs the proper docking of ISGs to the PM, consequently affecting insulin secretion. Furthermore, the higher ISG numbers observed in KCNH6-βKO β cells compared to control β cells can be attributed to the accumulation of secretory granules that are unable to undergo exocytosis due to the impaired docking process (Fig. 4F–I). We further confirmed that the mRNA expression levels of insulin biosynthesis-related genes in islets from control and KCNH6-βKO mice were not changed (Figure S4). These findings shed light on the crucial role of KCNH6 in regulating ISG docking to the PM to replenish depleted pools with glucose stimulation, highlighting its significance in the insulin secretion process and providing valuable insights into the underlying mechanisms of β cell dysfunction associated with KCNH6 deficiency.

### KCNH6 interacted with Munc18-1 in murine pancreatic β cells

**Fig. 5:**
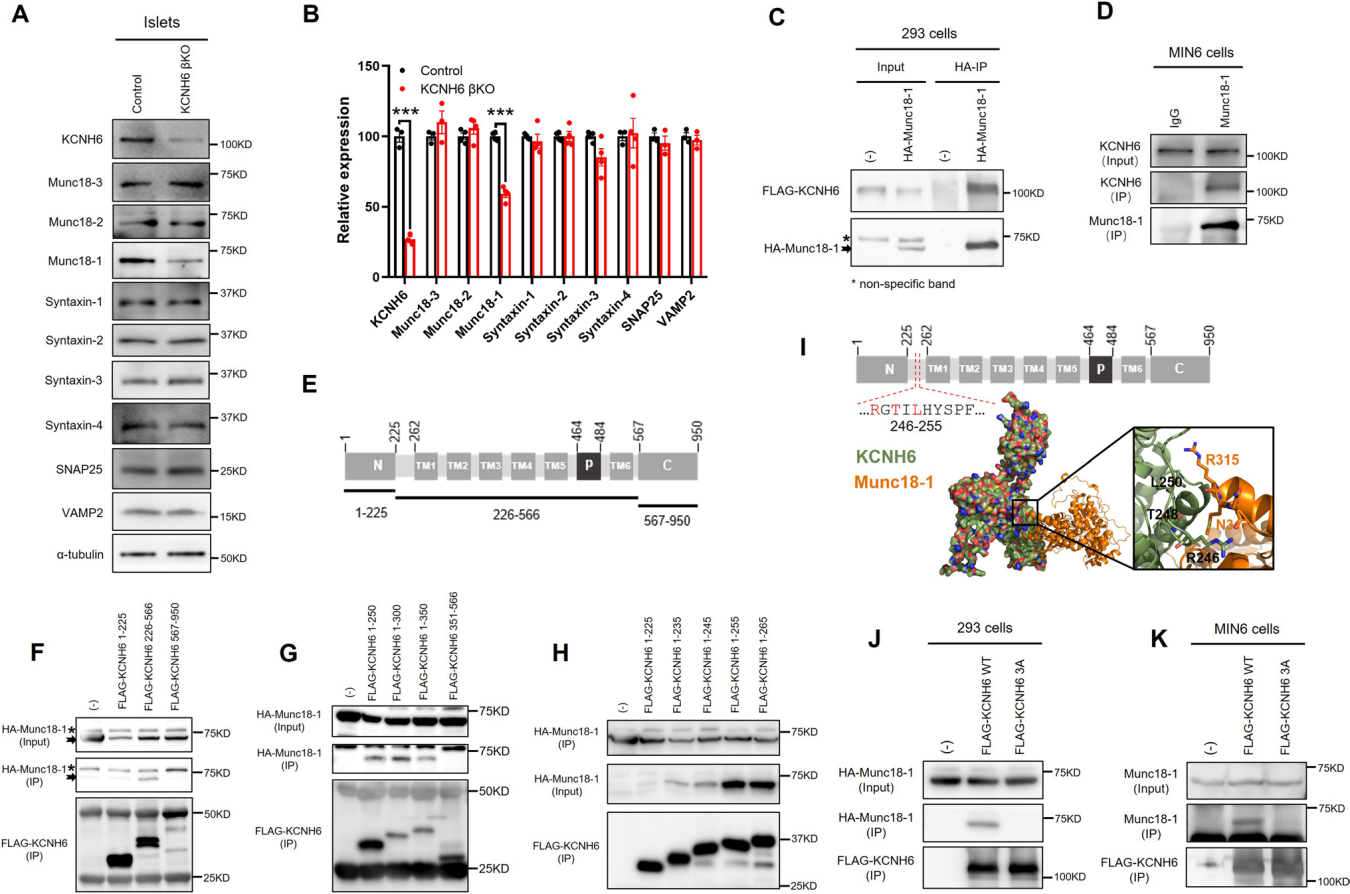
KCNH6 bound with Munc18-1 in murine pancreatic β cells. **A** Expression of insulin secretion-related proteins in pancreatic islets of control and KCNH6-βKO mice as detected by western blot analysis. **B** Statistical analysis of grayscale intensity values for protein expression levels in **A** (*n* = 3). **C** HEK293A cells were transfected to co-express HA-Munc18-1 and FLAG-KCNH6. The cell lysates were incubated with anti-HA beads, and the bound proteins and 1/50 volume of the reaction mixture were analyzed by immunoblotting with anti-HA or anti-FLAG antibodies. The extra band (asterisk) is the non-specific band. **D** MIN6 cells underwent immunoprecipitation with anti-Munc18-1 antibody or control IgG for checking endogenous interaction between Munc18-1 and KCNH6. The immunoprecipitates, as well as 1:20 of the original lysates, were immunoblotted with anti-KCNH6 and anti-Munc18-1 antibodies. The IP with anti-Munc18-1 antibody or anti-KCNH6 antibody, as well as 1/100 volume of the original lysates (Input), were analyzed by immunoblotting with the indicated antibodies. **E** Protein sequence diagram of one of the four subunits of KCNH6 channels. **F**–**H** HEK293A cells were transfected with HA-Munc18-1 and FLAG-KCNH6 truncated mutants. The cell lysates were incubated with anti-FLAG beads, and the bound proteins and 1/100 volume of the reaction mixture as input were analyzed by immunoblotting with anti-HA or anti-FLAG antibodies. The extra band (asterisk) is the non-specific band. **I** Molecular docking result of KCNH6 and Munc18-1 performed by HDOCK. KCNH6 is displayed as a surface in green. Munc18-1 is displayed as a cartoon in orange. In the zoomed-in view of the docking result, KCNH6 is shown as a green cartoon, and Munc18-1 is represented as an orange cartoon. Residues that are within a distance of less than 3.5 Å between KCNH6 and Munc18-1 (R246, T248 and L250) are illustrated as sticks. These residues are situated within the sequence corresponding to the N-terminus and the TM1 domain and indicated in the protein sequence diagram. **J** HEK293A cells were transfected with HA-Munc18-1 and FLAG-KCNH6-WT or FLAG-KCNH6 R246A/T248A/L250A (3A) mutants. The FLAG-immunoprecipitation were analyzed as in **B**. **K** MIN6 cells were infected with FLAG–KCNH6 WT or FLAG–KCNH6 3A mutant adenovirus. The FLAG-immunoprecipitation were analyzed as in **B**. Data shown are representative of three independent experiments for each; ***p < 0.001 by Student *t* test

To elucidate the molecular mechanisms underlying the impaired insulin exocytosis observed in KCNH6-βKO mice, we investigated the expression levels of key exocytotic proteins. We found that Munc18-1, the regulator of the SNAREs, was expressed at significantly lower levels in KCNH6-βKO islets (Fig. 5A and B). Because Munc18-1 is a critical regulator of insulin granule exocytosis in pancreatic β cells [16] and because protein expression levels often decline with loss of an interacting protein [24, 32], we hypothesized that KCNH6 interacts with Munc18-1. To explore this possibility, we expressed KCNH6 and Munc18-1 in 293A cells to check the exogenous binding activity between these two molecules. Co-immunoprecipitation results showed that KCNH6 interacts with Munc18-1 (Fig. 5C). Additionally, we confirmed that KCNH6 forms an endogenous complex with Munc18-1 in murine β cell line MIN6 cells (Fig. 5D). KCNH6 is a voltage-gated potassium channel protein with two non-transmembrane domains (residues 1–225 and residues 567–950) and one transmembrane domain (residues 226–566). The transmembrane domain comprises six segments, including a pore-forming loop essential for ion conduction and selectivity (Fig. 5E). To identify the binding regions of KCNH6 and Munc18-1, we co-expressed each domain with Munc18-1 in HEK293A cells. We observed that only the transmembrane domain formed a complex with Munc18-1 (Fig. 5F). To further explore the binding domain of KCNH6 with Munc18-1, we narrowed down the fragment of the transmembrane domain of KCNH6 and found that 226–300 residues could efficiently bind Munc18-1 (Fig. 5G). Further analysis revealed that the binding ability of residues 1–255 to Munc18-1 was significantly stronger compared to residues 1–245, suggesting that potential binding sites exist between residues 246–255 (Fig. 5H). To identify the specific amino acid residues in KCNH6 that are involved in the interaction with Munc18-1, we utilized the HDOCK web server to predict the protein–protein binding sites [33]. R246, T248, and L250 in KCNH6 were identified as the most probable binding site (Fig. 5I). According to this prediction, R246A/T248A/L250A (3A) triple mutant of KCNH6 was generated. We first proved that the 3A mutant diminished binding activity to exogenous Munc18-1 in transfected 293A cells (Fig. 5J). Then, we confirmed that the 3A mutant lost binding activity to endogenous Munc18-1 in MIN6 cells (Fig. 5K). Thus, the R246A/T248A/L250A mutant of KCNH6 specifically loses binding activity to Munc18-1.

**Fig. 6:**
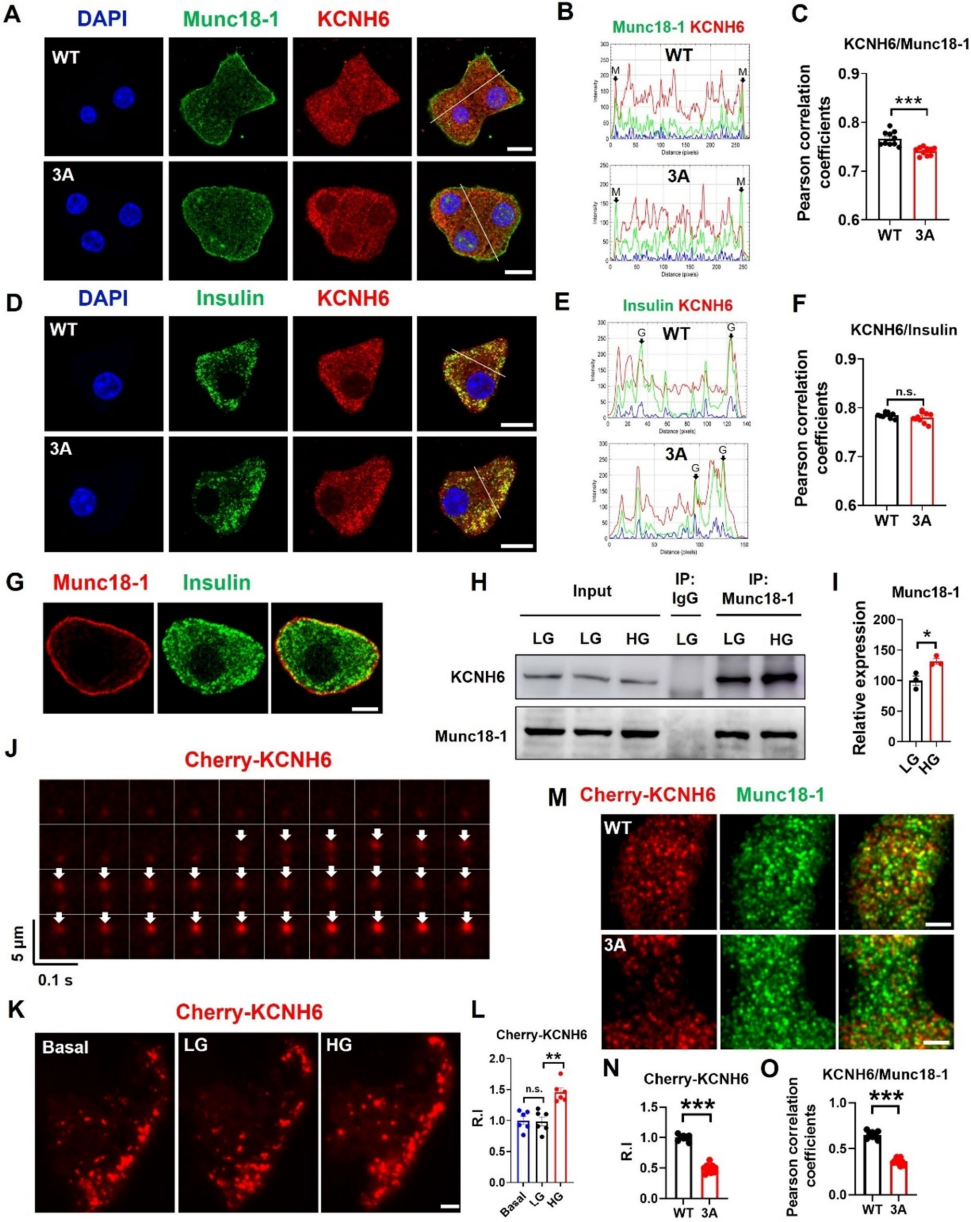
KCNH6 move to plasma membrane after glucose stimulation. **A** KCNH6-βKO pancreatic β-cells were infected with adenoviruses encoding WT or 3A mutant KCNH6. They were coimmunostained with mouse anti-Munc18-1 antibodies and rabbit anti-KCNH6 antibodies. Bar: 10 μm. **B** Fluorescent intensity profiles along the indicated line of KCNH6 and Munc18-1 are shown at right. Arrows indicated the membrane (M) regions. **C** Colocalization was quantified by Pearson’s correlation coefficient (*n* = 10 cells from 2 mice each). **D** KCNH6-βKO pancreatic β-cells were infected with adenoviruses encoding WT or 3A mutant KCNH6. They were coimmunostained with mouse anti-insulin antibodies and rabbit anti-KCNH6 antibodies. Bar: 10 μm. **E** Fluorescent intensity profiles along the indicated line of KCNH6 and insulin are shown at right. Arrows indicated the granule (G) regions. **F** Colocalization was quantified by Pearson’s correlation coefficient (*n* = 10 cells from 2 mice each). **G** Distribution of Munc18-1 and insulin granules was examined using confocal fluorescence microscopy. Bar: 10 μm. **H** Immunoprecipitation (IP) and immunoblotting were performed to examine the endogenous interaction between Munc18-1 and KCNH6 in MIN6 cells under different glucose concentrations (LG or HG). The cell lysates underwent IP with anti-Munc18-1 antibody, followed by immunoblotting with anti-Munc18-1 and anti-KCNH6 antibodies. **I** Statistical analysis of grayscale intensity values for protein expression levels in **H** (*n* = 3). *p < 0.05 by Student *t* test. **J**–**L** KCNH6-βKO pancreatic β-cells expressing Cherry-KCNH6 were incubated with 2.8 mmol/L glucose KRBB (LG) for 30 min, then were stimulated with 16.7 mmol/l glucose KRBB (HG). **J** The time-lapse fluorescence profiles of Cherry-KCNH6 (red) upon docking were monitored by TIRFM, white arrows indicated the docking events of Cherry-KCNH6. **K** KCNH6 null β-cells expressing Cherry-KCNH6 were incubated with LG for 30 min followed by HG for another 30 min, and TIRFM images of the same β-cells under LG and HG conditions respectively. Bar: 5 μm. **L** Relative fluorescence-intensity of (K) (*n* = 6). **p < 0.01 by one-way ANOVA. n.s. means not significant. **M** KCNH6 null β-cells expressing Cherry-KCNH6 WT or 3A mutant were analyzed by TIRFM after coimmunostaining with Munc18-1 and RFP antibodies. Bar: 5 μm. **N** Relative fluorescence-intensity of Cherry-KCNH6 (*n* = 10 from 3 mice each). **O** Colocalization was quantified by Pearson’s correlation coefficient (*n* = 10 cells from 3 mice each). *p < 0.05, **p < 0.01, ***p < 0.001 by Student *t* test

### KCNH6 translocated to the plasma membrane upon glucose stimulation

To detect the distribution of KCNH6 WT or 3A mutant and Munc18-1 or ISGs, immunostaining revealed that the 3A mutant expressed in KCNH6 null β cells did not colocalize with Munc18-1 along the plasma membrane (Fig. 6A–C) while the colocalization between KCNH6 3A mutant and insulin remained unaffected (Fig. 6D–F), which indicated that the KCNH6 3A mutant destroyed Munc18-1 binding activity on the plasma membrane without affecting its localization on ISGs. In addition, immunostaining revealed that Munc18-1 was mainly localized on the plasma membrane which was similar as described elsewhere [34]. We checked again for the distribution of Munc18-1 in murine pancreatic β cells with ISGs, immunofluorescence image showed that Munc18-1 was mainly expressed and colocalized with insulin on the plasma membrane (Fig. 6G). Next, we checked binding affinity between KCNH6 and Munc18-1 in MIN6 cells and found that it was enhanced following high glucose stimulation (Fig. 6H and I) since Munc18-1 on the plasma membrane is essential for ISG docking and fusion [16, 21, 34, 35], we speculated that KCNH6 positive granules to reach Munc18-1 on the plasma membrane for regulating ISG docking and fusion after glucose stimulation. To confirm our speculation, we examined the dynamic expression of KCNH6 on the plasma membrane with or without high glucose stimulation by TIRFM. We found that KCNH6 translocated from the cell interior to the plasma membrane (Fig. 6J) and this phenomenon was only occurred after high glucose stimulation but not low glucose stimulation, because the fluorescent intensity of KCNH6 from TIRFM analysis only increased after incubation of high glucose, but not changed under low glucose condition (Fig. 6L). Finally, we checked colocalization of KCNH6 and Munc18-1 on the plasma membrane after glucose stimulation by TIRFM analysis. KCNH6 colocalized with Munc18-1 on the plasma membrane, however, expression of KCNH6 3A mutant on the plasma membrane was very low and did not colocalized with Munc18-1 (Fig. 6M–O), which indicated KCNH6 3A mutant failed to dock to the plasma membrane because of disruption of binding with Munc18-1. These findings suggested that KCNH6 translocated to the plasma membrane upon glucose stimulation and docking to the plasma membrane through interaction with Munc18-1.

### KCNH6 3A mutant failed to rescue impaired insulin exocytosis from KCNH6 deficient β cells

**Fig. 7:**
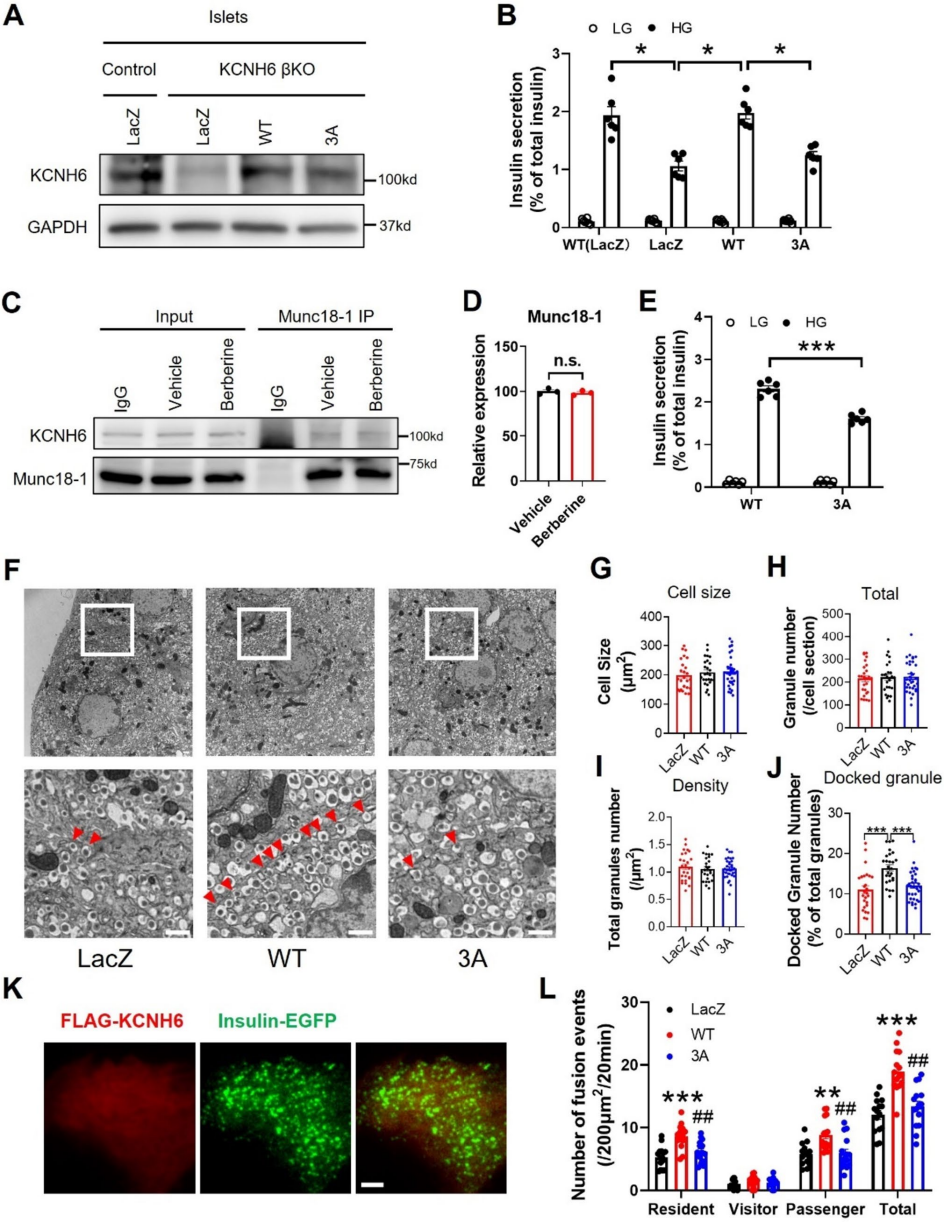
KCNH6 3A mutant failed to rescue impaired insulin exocytosis from KCNH6 deficient β cells. **A** The control and KCNH6-βKO islets were infected with adenoviruses encoding LacZ, KCNH6 WT or KCNH6 3A mutant. After a 1-h infection, the islets were rinsed and incubated for 48 h at 37 °C. The protein levels of KCNH6-3A were matched to those of KCNH6-WT expressed in KCNH6-βKO islets. **B** The control and KCNH6-βKO islets were infected with adenoviruses with the condition described in **A**. The islets were preincubated in 2.8 mmol/l glucose KRBB for 1 h and were incubated in 2.8 mmol/l low glucose (LG) or 16.7 mmol/l high glucose (HG) buffer for 1 h. Insulin levels secreted in the media and left in the cell lysates were measured, and their ratios are shown (*n* = 6 from six mice). *p < 0.05 by one-way ANOVA. **C** Immunoprecipitation (IP) and immunoblotting were performed to examine the endogenous interaction between Munc18-1 and KCNH6 in MIN6 cells after incubation with 10 μM Berberine for 1 h. The cell lysates underwent IP with anti-Munc18-1 antibody, followed by immunoblotting with anti-Munc18-1 and anti-KCNH6 antibodies. **D** Statistical analysis of grayscale intensity values for protein expression levels in **C** (*n* = 3). n.s. means not significant. **E** KCNH6-βKO islets expressing KCNH6 WT or 3A mutant were stimulated with HG for 60 min after incubation with 10 μM Berberine or vehicle for 1 h (*n* = 6 cells from 3 mice each). ***p < 0.001 by Student *t* test. **F** Insulin granule morphology in Control and KCNH6-βKO β cells under 16.7 mmol/l glucose KRBB condition (*n* = 26 for LacZ from 3 mice; *n* = 26 for WT from 3 mice; *n* = 33 for 3A from 3 mice). Squares in lower panels are shown at higher magnification in upper panels. White arrowheads indicate the granules of the reserve pool, whereas red arrows indicate the granules of the docked pool. Bar, 1 μm. **G–I** Cell size (G), total number of granules (**H**), and average granule density (**I**) in control and KCNH6-βKO β cells under HG condition. **J** T The percentage of docked granules to total granules in WT and KCNH6-βKO β cells after HG stimulation. A distance < 200 nm from the center of the granule to the plasma membrane was considered to be a docked granule. ***p < 0.01 by one-way ANOVA. **K** TIRFM image of living β-cells from KCNH6-βKO mice co-expressing FLAG-KCNH6/mCherry and Insulin-EGFP. Bar: 5 μm. **D** TIRF microscopic analysis of insulin granule exocytosis was performed as in Fig. 4 in KCNH6-βKO β cells co-expressing Insulin-EGFP and LacZ, KCNH6-WT or KCNH6-3A (R246A/T248A /L250A) mutant (*n* = 15 cells from five mice each). **p < 0.01, ***p < 0.001 vs LacZ, ^##^*p* < 0.01 vs WT by one-way ANOVA

Since KCNH6 is a Kv channel, to confirm whether the KCNH6 3A mutation affects its ion channel function, we first conducted KCNH6 current recordings in HKE293 cells transfected with either the wild-type KCNH6 (KCNH6 WT) or the KCNH6 3A mutant (KCNH6 3A). We found that there was no significant difference in the KCNH6 currents observed between KCNH6 WT and KCNH6 3A (Figure S5), indicating that the KCNH6 3A mutant retained its electrical function. To investigate whether the interaction with Munc18-1 is important for KCNH6’s promotion of insulin granule exocytosis, we performed rescue experiments. We introduced wild-type or 3A mutant KCNH6, as well as control LacZ protein, into KCNH6-βKO islets to match the level of endogenous KCNH6 in wild-type islets (Fig. 7A). Our results showed that the wild-type KCNH6 restored GSIS to levels comparable to those in control LacZ-expressing islets, while the 3A mutant failed to restore insulin secretion (Fig. 7B). We previously identified Berberine was a selective inhibitor of KCNH6 currents, to better understand whether the KCNH6/Munc18-1 complex promoting insulin secretion was independent of its electrical function, we analyzed GSIS from islets treated with Berberine to block KCNH6 current. We found that Berberine did not affec binding activity between KCNH6 and Munc18-1 in β cells (Fig. 7C and D), KCNH6 3A mutant showed decreased GSIS compared with that of KCNH6 WT expressed islets (Fig. 7E), which indicated that KCNH6 promoted GSIS through interaction with Munc18-1 independent of its electrical function. In addition, overexpression of KCNH6 WT or KCNH6 3A mutant in MIN6 cells showed similar results on GSIS as in islets (Figure S6), since KCNH6 3A mutant unaffected KCNH6 currents, this result again suggested that KCNH6 promoted GSIS through interaction with Munc18-1 independent of its electrical function. We then performed rescue experiments to examine whether defects in docking ability from KCNH6-deficient β cells after glucose stimulation are restored by the expression of exogenous KCNH6 under EM (Fig. 7F–J). We found that KCNH6 WT restored impaired docked ISGs in the KCNH6-deficient β cells, in contrast to the expression of control LacZ (Fig. 7F and J). Importantly, the restoration of docked ISGs was not seen by the expression of the KCNH6 3A mutant, which is specifically defective in binding to Munc18-1 (Fig. 5). This result suggested that KCNH6 promoted the docking of ISGs to the plasma membrane after glucose stimulation through interaction with Munc18-1. Finally, to obtain specific evidence on ISGs fusion, we conducted rescue experiments of insulin exocytosis in monolayer β-cells under TIRFM. KCNH6 WT or 3A mutant were introduced in KCNH6 null β-cells (Fig. 7K) and were stimulated with high glucose. Our findings demonstrated that KCNH6-βKO β-cells expressing KCNH6 WT exhibited a significant increase in the number of resident and passenger-type exocytic events in response to glucose stimulation. In contrast, the introduction of the KCNH6 3A did not result in such an increase (Fig. 7L). These findings suggested that KCNH6 regulates insulin exocytosis through interaction with Munc18-1 independently of its electrical function.

## Discussion

**Fig. 8:**
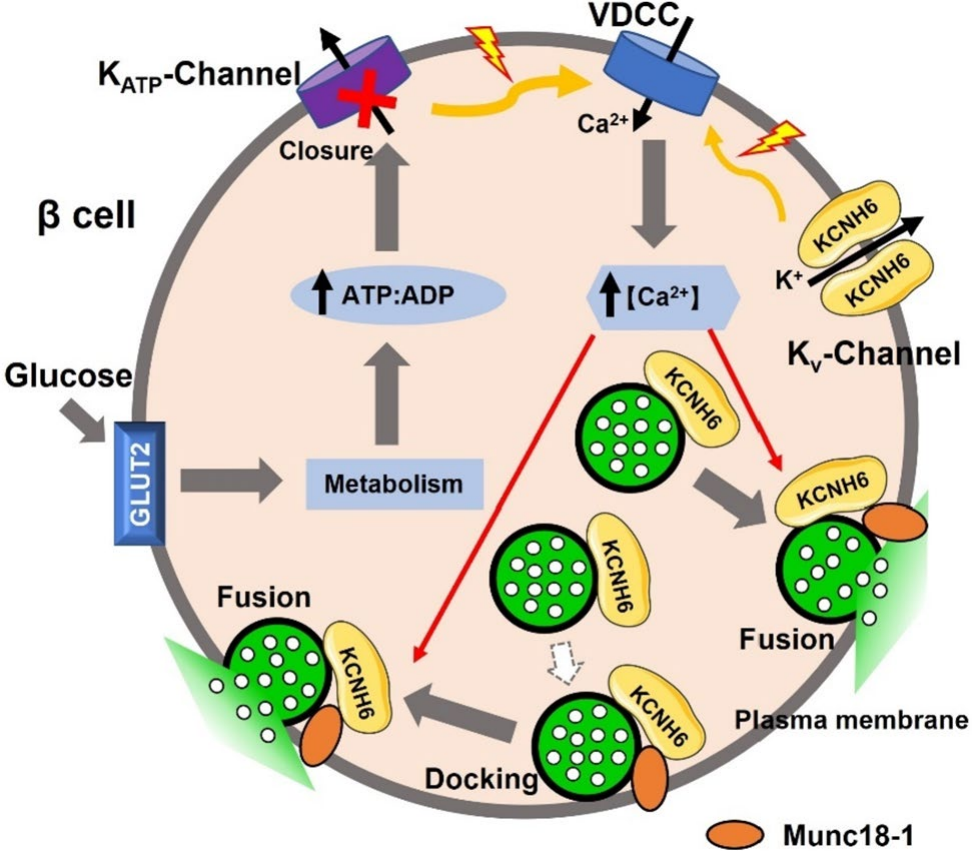
KCNH6 promoted insulin exocytosis through interaction with Munc18-1 in pancreatic β cells. Proposed model of KCNH6 promoting insulin exocytosis through the interaction of Munc18-1 in β cells. KCNH6 was localized on insulin secretory granules and mediated their docking and fusion to the plasma membrane after glucose stimulation through interaction with Munc18-1, which is independent of its electrical function

In this study, we provided evidence that KCNH6 directly promoted insulin exocytosis through its interaction with Munc18-1. KCNH6 was localized on insulin granules and translocated to the plasma membrane after glucose stimulation, and then interacted with Munc18-1, the SNARE binding partner, to induce subsequent docking and fusion of insulin granules from the plasma membrane (Fig. 8). Disrupting this native KCNH6-Munc18-1 interaction with a KCNH6 3A mutant leads to impaired glucose-stimulated insulin secretion. However, restoring KCNH6 expression can rescue this deficiency in insulin secretion. These findings indicate that KCNH6 have additional effects on insulin secretion beyond its electrical function.

We demonstrated KCNH6 was associated with ISG exocytosis (Fig. 2G) and played a direct role in insulin exocytosis of biphasic GSIS, as depletion of KCNH6 in pancreatic islets inhibited first- and second-phase GSIS (Fig. 1G–I) and, on TIRFM, was shown to be attributed to a reduction of passengers (undocked ISGs) [24] in first- and second-phase GSIS (Fig. 3D–H) and reduction of residents (predocked ISGs) [24] in first-phase GSIS. KCNH6 augments insulin granule exocytosis in β-cells through its interaction with Munc18-1. Firstly, Munc18-1 as a SNARE binding partner has been proven to regulate secretory vesicles docking and fusion [16, 35]. Secondly, triggering release after glucose stimulation enhanced KCNH6 binding to Munc18-1 (Fig. 6H–I). Thirdly, disruption of the Munc18-1 binding ability by the KCNH6 3A mutant almost completely abolished KCNH6-dependent facilitation of exocytosis (Fig. 7L). Thus, KCNH6-Munc18-1 complex played an important role in insulin exocytosis. Further work will be required to full elucidate the molecular mechanism of ISG fusion especially for passenger-type fusion.

In addition to its role in insulin granule exocytosis, KCNH6-Munc18-1 complex was also important for ISGs from the cell interior to the plasma membrane to replenish reduced docking ISGs after glucose stimulation, which are required after high-frequency or sustained stimulation. We showed that KCNH6-deficient caused a docking defect of ISGs from the cell interior to the PM after a 30-min glucose stimulation (Fig. 4). This resulted in a reduced passenger type of fusion and consequently reduced capacity to sustain second-phase GSIS. However, at basal condition, ISGs in KCNH6-null β-cells were able to eventually get to the PM, explaining the similar number of docked ISGs compared to control cells. Our data showed KCNH6 3A mutant failed to restore a reduced number of docked ISGs after glucose stimulation (Fig. 7F–J), indicating that this role in ISG docking to the PM through interaction with Munc18-1 since Munc18-1 was found to regulate secretory vesicles replenishment [36, 37]. Thus, KCNH6 binding with Munc18-1 might act in concert to replenish insulin ISGs to supply the RRPs after glucose-stimulated depletion. This could partially explain why the depletion of KCNH6 had such significant effects on passenger-type ISGs. Taken together, our results support a major role for KCNH6 in docking ISGs to the PM required to sustain secretion with continued stimulation.

Kv channels were purported to account for the repolarization of pancreatic islet β-cell action potentials, and this has long been believed to be its major contribution to the regulation of insulin secretion. Among likely contributors, the KCNH6 has been found as a major Kv currents in rodent and human β cells [5], although one or more other channels also contribute in rodents and humans [38, 39]. Inhibition or knockout of β cell KCNH6 prolongs the action potential, increases intracellular Ca^2+^ and insulin secretion of rodent islets [5, 6]. This suggests that KCNH6 acts as a negative regulator of insulin secretion through its electrical function. However, we found that KCNH6 was associated with insulin granules exocytosis (Fig. 2G), increasing KCNH6 enhances GSIS in MIN6 cells and loss of KCNH6 induced impaired abilities of docking and fusion from single β cells after glucose stimulation, suggesting that KCNH6 can also have a positive regulatory effect on insulin secretion. Further, we demonstrated that KCNH6 interacts with exocytotic SNARE proteins binding partner Munc18-1, and colocalizes with it around the plasma membrane, KCNH6 promoted ISG docking and fusion in β cells due to an interaction with Munc18-1 and this effect is lost upon disruption of KCNH6-Munc18-1 binding (Fig. 7). The ion-conducting and non-conducting roles of KCNH6 in beta cells do not interfere with each other. As a repolarizing potassium channel, antagonizing KCNH6 enhances insulin secretion in a Ca^2+^-dependent manner. Concurrently, KCNH6 also enhances insulin secretion independently of its electrical function, through its interaction with Munc18-1. This was confirmed by conducting a rescue experiment. The KCNH6 3A mutant which still keeps electrical function failed to restore insulin secretion. However,  Berberine, a selective inhibitor of KCNH6, did not impact the interaction between KCNH6 and Munc18-1 (Fig. 7C–D) and remained capable of promoting insulin secretion [6]. Meanwhile, after the closure of KCNH6 channels with Berberine, rescue 3A mutant in KCNH6 βKO pancreatic islets showed impaired GSIS. These findings suggested that KCNH6 played multiple roles in the regulation of insulin secretion, for both regulation of electrical function and modulation of exocytosis.

Finally, it should be noted that our previous data showed that β cells from the early age of KCNH6 GKO mice showed enhanced electrical activity, Ca^2+^ responses and insulin secretion [5], which agrees with the role of KCNH6 as a negative modulator of insulin secretion dependent on its electrical function. However, we did not observe increased insulin secretion in young KCNH6 βKO mice (Fig. 1 and Figure S2). This does not imply that KCNH6 operates differently in KCNH6 GKO mice compared to KCNH6 βKO mice. Firstly, similar to other potassium channel knockout mice, the hyperinsulinemic phase in KCNH6 βKO mice are notably brief and rapidly transitioning to a reduction in insulin secretion within a few days after birth [40], which may pose challenges in accurately capturing the hyperinsulinemic phase in the KCNH6-βKO mice. Secondly, KCNH6 βKO islets means KCNH6 β-cell specific deficient islet which is completely different from KCNH6 GKO islets lacking KCNH6 in all kinds of cells in islets, since KCNH6 expression is not restricted to the β cell, differences in insulin secretion between KCNH6 GKO and KCNH6 βKO mice could be due to KCNH6 deletion in other cell types of islets and compensatory changes at these sites. Pancreatic α cells play a crucial role in regulating insulin secretion [41, 42]. We found that KCNH6 is expressed in α cells (Fig. 1B), which may modulate insulin release by influencing glucagon secretion, potentially contributing to the divergent insulin secretion phenotypes observed in KCNH6 GKO and KCNH6 βKO mice. Thirdly, we recently found that KCNH6 also regulates incretin secretion, the loss of KCNH6 enhanced incretin secretion, which might compensate for impaired insulin secretion from KCNH6 GKO mice before 14 weeks. Thus, KCNH6 plays multiple roles in the regulation of insulin secretion, serving both as a regulator of electrical function and in the direct modulation of exocytosis. Interestingly, the KCNH6 βKO mice phenotype are in part consistent and partly inconsistent with human carriers of the risk allele of KCNH6 [5]. This could be because heterozygous mutations in KCNH6 don't impact its role in regulating exocytosis in human pancreatic islet β cells. In future studies, we will gather additional clinical data and samples to further investigate the function of KCNH6 in human islets.

In conclusion, we demonstrated that KCNH6 facilitates β cell exocytotic responses by binding to Munc18-1, which is independent of its conventional electrical role. Disruption of interaction of KCNH6 and Munc18-1 impairs glucose-dependent replenishment and reduces glucose-stimulated biphasic exocytosis of ISGs. These findings provide a promising and novel avenue for enhancing our understanding of the KCNH6 channel in diabetes mechanisms. Further research will be required to determine the molecular mechanism of KCNH6 in insulin granule trafficking and in the fusion process itself.

### Supplementary Information

Below is the link to the electronic supplementary material.

Supplementary file1 (DOCX 756 KB)

## Data Availability

All data are contained within the article.
