# Supplementary material for: KCNH6 channel promotes insulin exocytosis via interaction with Munc18-1 independent of electrophysiological processes
Source: Cell Mol Life Sci. 2024 Feb 13;81(1):86. doi: 10.1007/s00018-024-05134-1 (PMC10864572; doi:10.1007/s00018-024-05134-1)
Supplement: Supplementary file 1 — Supplementary file1 (DOCX 756 KB) [file 18_2024_5134_MOESM1_ESM.docx]

**Supporting Information for:**

**KCNH6 channel promotes insulin exocytosis via interaction with Munc18-1 independent of electrophysiological processes**

Hao Wang^1,2,*,🖂^ Qi Li^1,2,*^ Ying-Chao Yuan^1^, Xue-Chun Han^1^, Yong-Ting Cao^3^ and Jin-Kui Yang^1,2,🖂^

^1^Beijing Key Laboratory of Diabetes Research and Care, Department of Endocrinology and Metabolism, Beijing Diabetes Institute, Beijing Tongren Hospital, Capital Medical University, Beijing 100730, China.

^2^Laboratory for Clinical Medicine, Capital Medical University, 100069, Beijing, China.

^3^Department of Endocrinology, Beijing Mentougou District Hospital, Beijing 102399, China.

*These authors contributed equally: Hao Wang and Qi Li

^🖂^Correspondence: Hao Wang (hwang@mail.ccmu.edu.cn) or Jin-Kui Yang (jkyang@ccmu.edu.cn)

**This file includes:**

Supplementary Table 1

Supplementary Table 2

Supplementary Table 3

Supplementary Figure 1

Supplementary Figure 2

Supplementary Figure 3

Supplementary Figure 4

Supplementary Figure 5

Supplementary Figure 6

**Supplementary Table 1. The sources of commercial reagents and the concentrations of the antibodies used for immunofluorescence (IF), immunoblotting (IB), or immunoprecipitation (IP)**

| **Antibody** | **Clone** | **Vendor** | **Catalog number** | **RRID** | **Dilution** | **Applica**  **-tion** |
| --- | --- | --- | --- | --- | --- | --- |
| anti-KCNH6 | Rabbit polyclonal | Our Lab |  |  | IB 1/1000; IF1/100 | IB, IF |
| Insulin | Mouse monoclonal | Cell Signaling Technology, Inc., MA, USA | 8138S | AB_10949314 | 1/500 | IF |
| Glucagon | Mouse monoclonal | Sigma-Aldrich, St.Louis, MO, USA | S2654 | AB_259852 | 1/200 | IF |
| FLAG | Rabbit polyclonal | Sigma-Aldrich, St.Louis, MO, USA | F7425 | AB_439687 | WB 1/5000; IF 1/500 | IB, IF |
| HA (hemagglutinin) | Rabbit polyclonal | MBL, Nagoya, Japan | 561 | AB_591839 | 1/1000 | IB |
| GAPDH (3H12) | Mouse monoclonal | MBL, Nagoya, Japan | M171-3 | AB_10597731 | 1/1000 | IB |
| β-actin | Mouse monoclonal | Sigma-Aldrich, St.Louis, MO, USA | A5316 | AB_476743 | 1/10000 | IB |
| α-tubulin | Mouse monoclonal | Sigma-Aldrich, St.Louis, MO, USA | T5168 | AB_477579 | 1/10000 | IB |
| Syntaxin-1  (HPC-1) | Mouse monoclonal | Sigma-Aldrich, St.Louis, MO, USA | S0664 | AB_477483 | 1/1000 | IB |
| Syntaxin-2 | Rabbit polyclonal | Synaptic Systems, Goettingen, Germany | 110 123 | AB_887849 | 1/1000 | IB |
| Syntaxin-3 | Rabbit polyclonal | Synaptic Systems, Goettingen, Germany | 110 033 | AB_887851 | 1/1000 | IB |
| Syntaxin-4 | Rabbit polyclonal | Merck KGaA, Darmstadt, Germany | AB5330 | AB_91042 | 1/1000 | IB |
| Rab27a | Rabbit polyclonal | Cell Signaling Technology, Inc., MA, USA | 69295S | AB_2799759 | IB 1/1000 | IB |
| VAMP2 | Rabbit polyclonal | Proteintech, Wuhan, BeiHu, China | 10135-1-AP | AB_2256918 | 1/1000 | IB |
| SNAP25 | Rabbit polyclonal | Proteintech, Wuhan, BeiHu, China | 14903-1-AP | AB_2192051 | 1/1000 | IB |
| Munc18-1 | Mouse monoclonal | BD BioScience, San Jose, CA, USA | 610336 | AB_397726 | 1/10000 | IB |
| Munc18-1 | Rabbit polyclonal | Proteintech, Wuhan, BeiHu, China | 11459-1-AP | AB_2196690 | 1μg  /sample | IP |
| Munc18-1 | Mouse monoclonal | Proteintech, Wuhan, BeiHu, China | 67137-1-Ig | AB_2882436 | 1/100 | IF |
| Munc18-2 | Rabbit polyclonal | LifeSpan BioSciences, Inc., Seattle, WA, USA | LS-B2356 | AB_2271157 | 1/1000 | IB |
| Munc18-3 | Rabbit polyclonal | Sigma-Aldrich, St.Louis, MO, USA | M7695 | AB_1080121 | 1/1000 | IB |
| Calnexin | Mouse monoclonal | Proteintech, Wuhan, BeiHu, China | 66903-1-Ig | AB_2882231 | 1/1000 | IB |
| Na^+^-K^+^-ATPase | Rabbit Monoclonal | Abcam, Cambridge, UK | ab76020 | AB_1310695 | 1:1000 | IB |
| Anti-FLAG M2 Affinity Gel | Rabbit polyclonal | Sigma-Aldrich, St.Louis, MO, USA | A2220 | AB_10063035 | 30 μl/sample | IP |
| Anti-HA Affinity Matrix | Rat monoclonal | Roche Diagnostics GmbH, Mannheim, Germany | 11815016001 | AB_390914 | 20 μl/sample | IP |
| Protein G Sepharose 4 Fast Flow |  | GE Healthcare Biosciences, Uppsala, Sweden | GE17-0618-01 |  | 40 μl/sample | IP |
| Pierce^TM^ ProteinA/G Magnetic beads |  | Invitrogen, Thermo Fisher Scientific, Waltham, MA, USA | 88802 |  | 50 μl/sample | IP |

**Supplemental Table 2. Primer for cloning and mutagenesis**

| Target | Primer | Sequence |
| --- | --- | --- |
| KCNH6 full length | Forward | GGTGTACAATGCCAGTCCGCAGGGGCCACGT |
|  | Reverse | GGGGATCCCTAACTCCTTGTGAATCCAGG |
| Munc18-1 full length | Forward | GCTAGCATGGCCCCCATTGGCCTCAAGGCGG |
|  | Reverse | CTCGAGTTAACTGCTTATTTCTTCATCTGTTTTA |
| KCNH6 fragment 1-225 | Forward | GGTGTACAATGCCAGTCCGCAGGGGCCACGT |
|  | Reverse | GGGGATCCCTAACTGAAAAGGTCACGCAG |
| KCNH6 fragment 226-566 | Forward | GGTGTACAGTCCTGTCCCTGGGTGCA |
|  | Reverse | GGGGATCCGTTCATGTCGATGCCATT |
| KCNH6 fragment 567-950 | Forward | GGTGTACAGCGGTGCTGAAAGGCTTC |
|  | Reverse | GGGGATCCCTAACTCCTTGTGAATCCAGG |
| KCNH6 fragment 1-250 | Reverse | GGGGATCCCTACAGAATGGTACCTCGGTG |
| KCNH6 fragment 1-300 | Reverse | GGGGATCCCTAGAGCGGGCTGCAGGTATA |
| KCNH6 fragment 1-350 | Reverse | GGGGATCCCTACATGTCAATGAGGAACCA |
| KCNH6 fragment 301-566 | Forward | GGTGTACAACCGTGGTGGACCTCATC |
| KCNH6 fragment 1-235 | Reverse | GGGGATCCCTATGGCAGCACATCTGCACC |
| KCNH6 fragment 1-245 | Reverse | GGGGATCCCTAGTGAATGCGCGGTGCCTG |
| KCNH6 fragment 1-255 | Reverse | GGGGATCCCTAGAAGGGGCTGTAGTGCAG |
| KCNH6 fragment 1-265 | Reverse | GGGGATCCCTAGAGCAAGATGAGCCAGTC |
| KCNH6 fragment R246A/T248A/L250A | Forward | CGCATTCACGCTGGTGCTATTGCTCACTACAGC |
|  | Reverse | GCTGTAGTGAGCAATAGCACCAGCGTGAATGCG |

**Supplemental Table 3. Primer for quantitative PCR**

| Target | Primer | Sequence |
| --- | --- | --- |
| Preproinsulin | Forward | CCCTGCTGGCCCTGCTCTT |
|  | Reverse | AGGTCTGAAGGTCACCTGCT |
| INS1 | Forward | CCTTCAGACCTTGGCGTTGG |
|  | Reverse | CGAGGTGGGCCTTAGTTGCA |
| INS2 | Forward | ACCCACAAGTGGCACAACTG |
|  | Reverse | AGGGGTAGGCTGGGTAGTG |
| GCG | Forward | GGCACATTCACCAGCGACTACA |
|  | Reverse | GCCCTCCAAGTAAGAACTCACATC |
| Somatostatin | Forward | ATCGTCCTGGCTTTGGGCGGTGTCA |
|  | Reverse | TCTGTCTGGTTGGGCTCGGACAGCAGCTCT |
| KCNH6 | Forward | CGTTTGTTGTCACACAGCTTCCTG |
|  | Reverse | TCCACAAAGCTGAGCGTGAACTG |
| KCNH2 | Forward | ATGGCTCAGATCCAGGCAGTTA |
|  | Reverse | CAAGGAGAGCGGTCAGGTAATG |
| KCNH7 | Forward | AACAGTGCAAATGACGCTGACGAC |
|  | Reverse | TCTTCACCATGGAGTCTGGTTGCT |
| KCNJ11 | Forward | GACATCCCCATGGAGAATGG |
|  | Reverse | TCGATGACGTGGTAGATGATGAG |
| Kv2.1 | Forward | CGTCATCGCCATCTCTCATG |
|  | Reverse | CAGCCCACTCTCTCACTAGCAA |
| Pdx-1 | Forward | CGGCTGAGCAAGCTAAGGTT |
|  | Reverse | TGGAAGAAGCGCTCTCTTTGA |
| Pax6 | Forward | AGTGTCTACCAGCCAATCCC |
|  | Reverse | GCAGGTTGTTTGCCATGGTG |
| NeuroD | Forward | CCTTTTAACAACAGGAAGTGG |
|  | Reverse | TAGACAGTTTCTGGGTCTTG |
| MafA | Forward | GTGGTGATGGTGGTGATGGTGA |
|  | Reverse | GGAGGATCTGTACTGGATGAGC |
| GAPDH | Forward | TCAACAGCAACTCCCACTCTTCCA |
|  | Reverse | ACCCTGTTGCTGTAGCCGTATTCA |
| Rplp0/36B4 | Forward | GGCCCTGCACTCTCGCTTTC |
|  | Reverse | TGCCAGGACGCGCTTGT |

**Supplementary Figures**


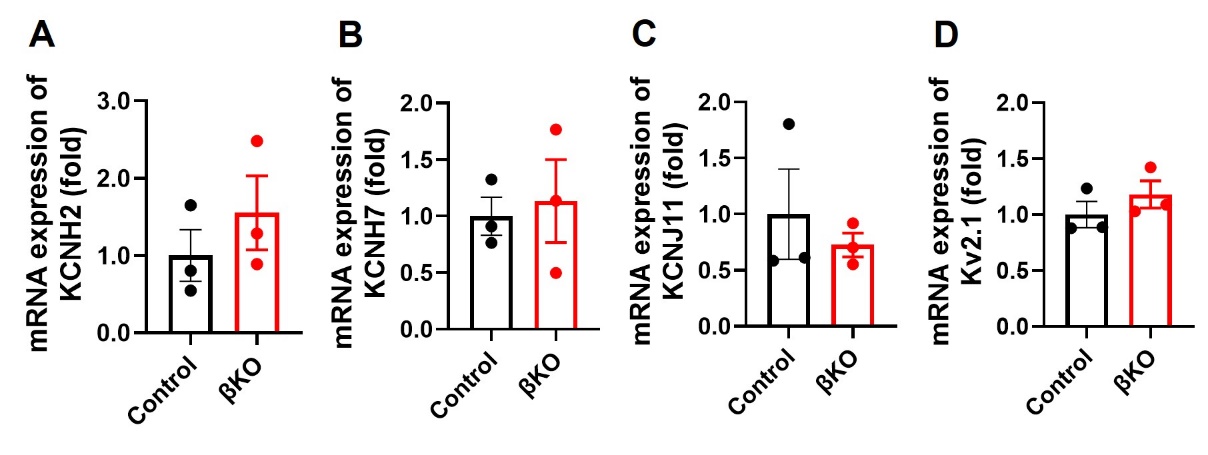


**Figure S1. mRNA expression of Kv channels in KCNH6 deficient islets.**

(A-D) mRNA expression levels of several K_V_ channels in islets from control and KCNH6-βKO mice. The statistical significance of differences between means was assessed by Student *t* test.


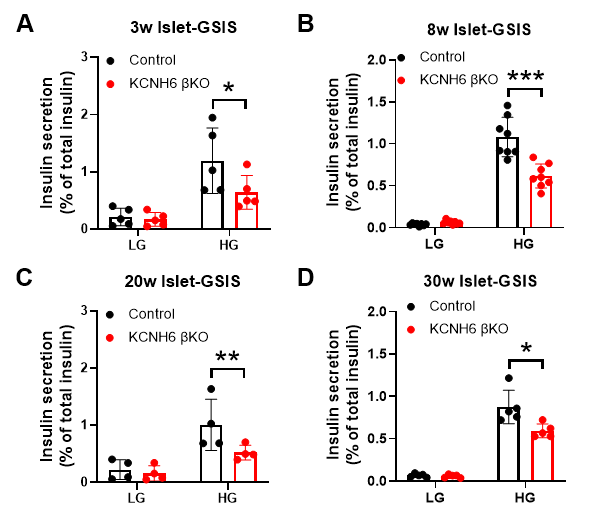


**Figure S2. Impaired GSIS from KCNH6 βKO islets was independent of age.**

(A-D) Glucose stimulated insulin secretion assays were conducted using islets isolated from control and KCNH6-βKO mice at different ages (A for 3-week-age, n = 5; B for 8-week-age, n = 8; C for 20-week-age, n = 4; D for 30-week-age, n = 5). The statistical significance of differences between means was assessed by Student *t* test. *^*^p* < 0.05, *^**^p* < 0.01, *^***^p* < 0.001.


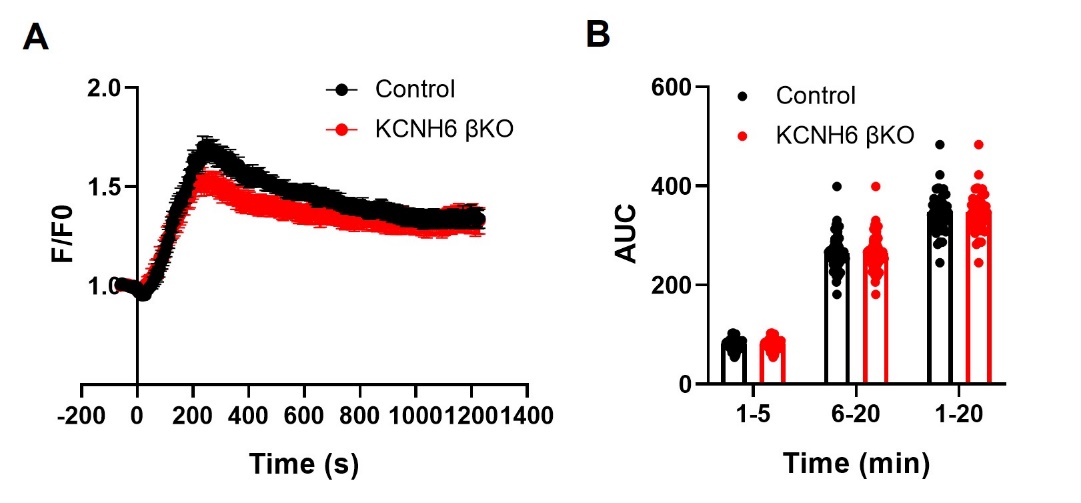


**Figure S3. KCNH6 deficient β cells showed no alteration of intracellular calcium concentration after glucose load.**

(A) Glucose-stimulated intracellular calcium level of pancreatic β cells isolated from control and KCNH6-βKO mice. (B) Total intracellular calcium level was calculated as the area under the curve (AUC) (n = 44). The statistical significance of differences between means was assessed by Student *t* test.


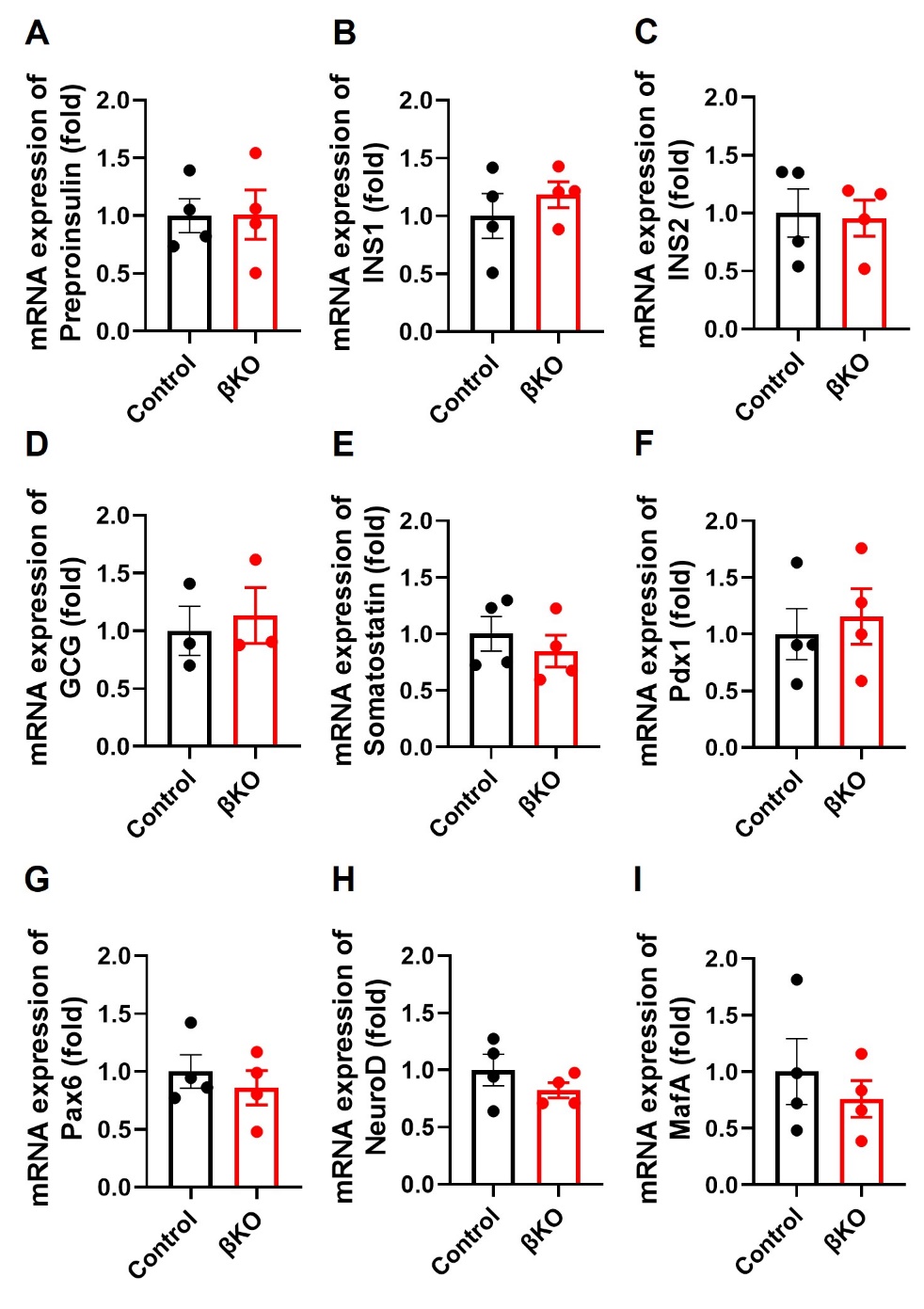


**Figure S4. Expression of insulin biosynthesis-related genes in KCNH6 deficient islets.**

(A-I) mRNA expression levels of insulin biosynthesis-related genes in islets from control and KCNH6-βKO mice (n = 3-4). The statistical significance of differences between means was assessed by Student *t* test.


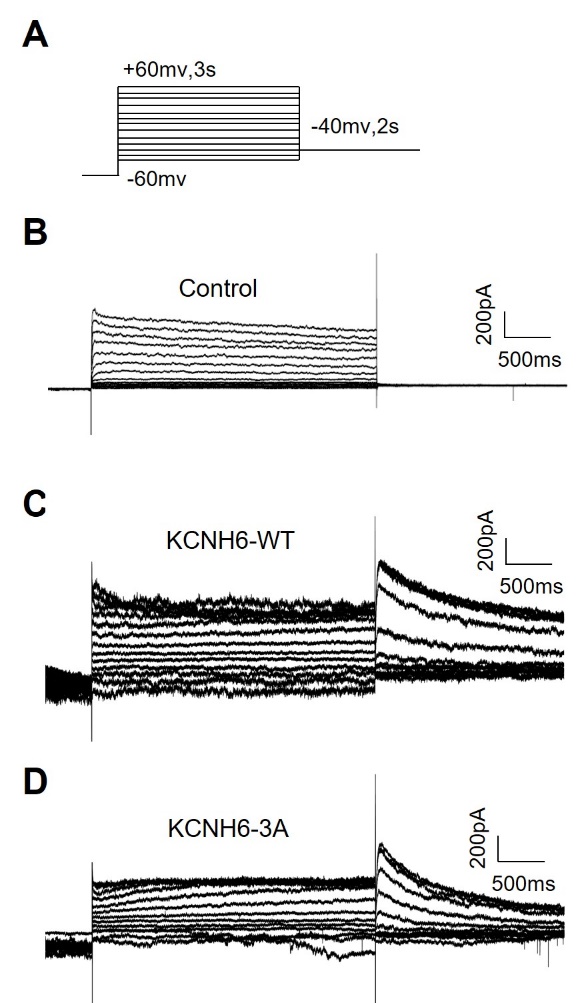


**Figure S5. KCNH6 3A mutant did not affect potassium current.**

(A-D) Representative whole-cell recordings of KCNH6 currents in the untransfected KCNH6-null HEK293T cells and KCNH6-transfected HEK293T cells. Untransfected KCNH6-null HEK293T cells (B) and transfected with either KCNH6 WT (C) or KCNH6 3A (D) were measured using voltage-clamp mode.


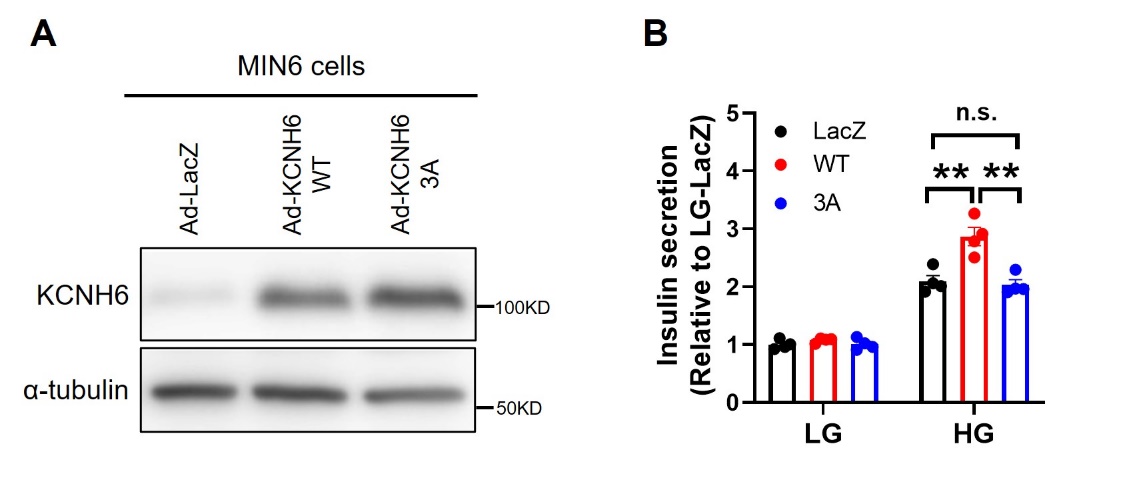


**Figure S6. KCNH6 3A mutant failed to enhance GSIS.**

(A) Immunoblotting analysis of MIN6 cells infected with adenoviruses encoding LacZ (control), KCNH6 WT and KCNH6 3A mutant. The expression of KCNH6 was detected using an anti-KCNH6 antibody. (B) The infected MIN6 cells were preincubated in 2.8 mmol/l glucose-containing KRBB for 1 h and were incubated in 2.8 mmol/l low glucose (LG) or 16.7 mmol/L high glucose (HG) buffer for 1 h. Secreted insulin and total insulin were measured (n = 4). The statistical significance of differences between means was assessed by one-way ANOVA. ***p* < 0.01, n.s. means not significant.
